# Supplementary material for: Aborted propagation of the Ethiopian rift caused by linkage with the Kenyan rift
Source: Nat Commun. 2019 Mar 21;10:1309. doi: 10.1038/s41467-019-09335-2 (PMC6428852; doi:10.1038/s41467-019-09335-2)
Supplement: Supplementary file 1 — Supplementary Information [file 41467_2019_9335_MOESM1_ESM.pdf]

Supplementary Information

Aborted propagation of the Ethiopian rift caused by linkage with the Kenyan rift

Corti et al.

Supplementary Note 1: Tectonics of the Turkana depression

The Turkana depression is a portion of the East African rift, a >3000km long aligned succession of adjacent individual tectonic basins resulting from the ongoing separation between the African and Somalian plates[1]. Plate divergence is occurring in a roughly E-W direction, at rates of a few millimetres per year[1]: the velocity of plate motion decreases from the northern Ethiopian rift (~5 mm yr^{-1}) southwards; extension at the latitude of Lake Turkana occurs at rates of ~3 mm yr^{-1} (ref. 1).

The Turkana depression is the region of interaction between the Ethiopian and the Kenyan rifts. In both rifts, extensional deformation gives rise to a typical narrow rift morphology, with large fault escarpments (with vertical displacement of >1 km) separating a roughly 80-100 km wide rift valley from uplifted plateaus. Conversely, faulting, earthquakes and Quaternary-Holocene volcanic centres in the Turkana depression are widespread over a width of more than 300 km (Supplementary Fig. 1). In addition, the Turkana region lacks the typical rift valley morphology dominated by large fault escarpments. Instead, extension results in numerous faults with limited vertical offset clustered within three main different sub-parallel domains of deformation[2]: (i) the Lake Turkana basin, (ii) the Kino Sogo fault belt and (iii) the Ririba rift (see main text).

The anomalous structural pattern and physiography of the Turkana depression is likely controlled by variations in the pre-rift structure of the lithosphere[3]. Geophysical data[4,5] provide evidence for significant crustal thinning beneath the NW-SE-trending Turkana depression. This thinning resulted from Mesozoic-Paleogene rift events, which created a system of NW-SE grabens (such as the Anza graben in Ethiopia, or the South Sudan rifts)[6] at a high angle to the Cenozoic Ethiopian/Kenyan rift valleys (Supplementary Fig. 1). The inherited thin crust related to these Mesozoic-Paleogene events was suggested to cause the low topography of the region with respect to the surrounding uplifted plateaus[4]. Numerical and physical modelling of rifting indicate that the transversal pre-existing heterogeneity with thinned crust and lithosphere also likely controlled the transition from localized deformation in the rift valley domains to the diffuse deformation in the Turkana depression, therefore influencing rift linkage in the region[3]. Similarly, pre-existing inherited structures have been suggested to have largely controlled the pattern of faulting at the scale of individual faults and fault arrays[7,8].

The Oligocene-Recent evolution, summarized in Supplementary Fig. 1, is characterised by the early activation of different N-S trending basins (e.g., Lotikipi, Gatome, Lokichar) West of Lake Turkana[9,10,11,12,13,14,15] (Supplementary Fig. 1). These basins started developing during the Oligocene-Early Miocene, after the earliest manifestation of flood volcanism at ~45 Ma in South Ethiopia[2]. The activity of the westernmost basins (Lotikipi, Gatome) ceased during the Middle Miocene. During the Early-Middle Miocene, extension started in some of the basins related to the Kenyan rift system (e.g., Samburu, Turkana, Kerio), and in the Chew Bahir basin. This activity was contemporaneous with a first, minor phase of extensional deformation in the Southern Main Ethiopian rift (Supplementary Fig. 1). Major phases of extension started in the Late Miocene in the Gofa Province of South Ethiopia and in the Southern Main Ethiopian rift (Abaya, Chamo, Galana basins and Amaro Horst region). During the Pliocene-Quaternary, the volcano-tectonic activity focused in the Lake Turkana basin, now a direct continuation of the Kenya rift, whereas the extensional deformation

related to the Ethiopian rift have been suggested to have propagated southward into the Eastern Turkana depression to form the Ririba rift and the Hurri Hills and Marsabit volcanic lineaments (Supplementary Fig. 2).

Overall, a progressive eastward migration of Oligocene-Recent deformation within the Turkana depression (from early extension in the basins West of Lake Turkana to the domains of active deformation of Lake Turkana, Chew Bahir-Kino Sogo and Ririba), has been suggested[2] (Supplementary Fig. 2). This migration, together with a northward propagation of rifting in the Kenya rift and a southward propagation of the Main Ethiopian rift to the Ririba and Marsabit volcanic lineaments, has been considered to have contributed to the anomalous breadth of the zone of deformation in the region[2].

Supplementary Note 2: Analysis of maximum displacement (D)/length (L) profiles and influence of pre-existing basement structures on faulting

We have analysed the maximum displacement (D) and trace length (L) relationship for the major normal faults that bound the Ririba rift on its western margin (Supplementary Fig. 3). The D/L relations varies over different length scales depending on several factors. These include: (1) material property, (2) type of fault including position and direction of measurement, (3) earthquake rupture and slip/propagation history, (4) segmentation and linkage (evolution), and (5) reactivation of pre-existing structures (see ref. 16 and references therein). The D/L relations can be used to characterize the growth model of faults, which can be referred to two end-members: (1) the segment growth and linkage model, where initially isolated fault segments elongate and accumulate displacement via simple tip propagation before eventually linking to form a through-going fault system, and (2) the constant length model, in which fault lengths are rapidly established and, thereafter, growth is dominated by displacement accumulation with limited (if any) lateral propagation[17]. This latter growth model is directly applicable to reactivated fault systems in which fault lengths are inherited from underlying structures and established almost instantaneously on geological timescales[17]; the earliest phases of growth of reactivated faults are therefore characterized by a significant under-displaced nature. D-L curves from the boundary faults of the Ririba rift (Supplementary Fig. 4) are characterized by flat top profiles, low-end gradients and low D-L values, which are in agreement with the constant length model. This supports our hypothesis that the development of major faults in the Ririba rift is controlled by reactivation of old, large basement structures.

This is also confirmed by analysis of basement fabrics in the region, which show three main trends, oriented respectively NNW-SSE, NW-SE and NE-SW (Supplementary Fig. 5).

The NNW-SSE trend is well expressed in the surroundings of the Chew Bahir basin[7], where it has been shown to have exerted a strong control on the development of the major normal faults bounding the depression[18,19]. The NW-SE basement fabric parallels the orientation of the Mesozoic Anza graben and South Sudan rifts and diffusely characterise the area East of the Ririba rift and the Mega escarpment. The NE-SW fabric is represented by minor structures diffused throughout the region of interest and has been associated to the

existence of a large, 30 km-wide transversal fault zone (Buluk fault zone[7,8]) marking the southern termination of the Chew Bahir and Segen basins (Supplementary Fig. 5).

The different sets of faults characterizing the Ririba rift are sub-parallel to these basement fabrics (Supplementary Fig. 5). In particular, the main boundary faults are characterized by a striking similarity in orientation with the NNW-SSE fabric; similarly, the minor sets of faults giving rise to the complex angular patterns parallel the NNE-SSW, NE-SW or ENE-WSW basement trends. This parallelism, the highly variable orientations of the Ririba rift faults, which are typically not orthogonal to the extension direction, and the above observations on the D-L curves support a strong influence of the pre-existing structures on fault development, as also observed in the nearby Kino Sogo fault belt[7,8].

Supplementary Note 3: Chemical analysis of samples of volcanic rocks

Whole rock analyses were performed on 9 samples from the Dilo area, collected both from the Pliocene lava flows clearly cut by the main Ririba faults and from the products discordantly lying on these lava flows or directly sealing the faults (Supplementary Table 2). Results of this analysis show that samples from the faulted Pliocene lava pavement of the Ririba rift (MDZ4, 5 and 12) plot in the field of subalkaline basalts (Supplementary Fig. 8), while the products from the top of the sequence, and in some cases sealing the faults of the Ririba rift, plot in the fields of basanite/tephrite up to foidite and are similar in composition to the Quaternary products of the area (e.g., ref. 4). The minor compositional variability observed in the Quaternary products is possibly partially related to the presence of abundant (locally up 10-15% by volume) mantle xenoliths or xenocrysts, and to processes of fractional crystallization.

Zr/Nb ratios (Supplementary Fig. 9) confirm the presence of two compositionally distinct groups, with the Quaternary lavas showing a nearly constant low ratio of 3.2, close to the value of 3.5 suggested for primary magmas of basanite-phonolite trend of the area[20]. Conversely, Pliocene rocks show a higher Zr/Nb ratio (8-9) comparable to that observed from continental tholeiites[21].

Supplementary Note 4: Analysis of vent alignment

The results of the analysis of vent alignment (Supplementary Fig. 10) indicate an overall, NE-SW elongation of the Dilo-Dukana volcanic field, with a complex distribution of the vents at a more local scale. The main peaks of vent distribution are indeed at N10°-20°E, N40°-50°E, with a minor peak in distribution at N20°W. Overall, these results indicate that the majority of the vent alignments are not orthogonal to the regional, current E-W direction of extension and therefore do not directly respond to stresses controlled by plate motion. Instead, the overall elongation of the field, subparallel to a major pre-existing structure (the Buluk fault zone) hypothesized west of the Ririba rift[7,8] (see above Supplementary Note 2), is suggestive of a strong control exerted by inherited basement fabrics on the distribution of volcanic vents. This is supported by distribution of vents at local scale, which is controlled by the angular network of NNE-SSW, NE-SW or ENE-WSW structures that reactivate pre-existing fabrics, as discussed above.

Supplementary Note 5: Other geological and morphological evidence for fault inactivity and constraints on the timing of rift abandonment

Many different lines of evidence indicate a lack of Pleistocene deformation activity in the Ririba rift and surrounding areas. Apart from the Late Pleistocene large lava flows burying the faults, recent fault inactivity is confirmed by the presence on the two main faults of the rift of small drainages that locally incise the scarps and generate 100-200 m-long fans (Fig. 3; Supplementary Fig. 11). These small fans, made up of colluvium, have their apex upstream the fault scarp indicating that the sedimentary rate is higher than the slip rate of the faults. As a further confirmation, elsewhere the Ririba River channel is located in the middle of the half graben, not because the sediment input from the footwall is enough to fill the site of maximum subsidence[22], but because the river is not forced by the subsidence of the hangingwall to flow close the fault plane.

Analysis of satellite images indicates that the normal faults at the southern termination of the rift affect the Late Miocene-Pliocene Bulal lavas but are covered by the basal flows of the Hurri Hills edifice (Supplementary Fig. 12a). Similarly, faults of the complex, angular network affecting the basal lava platform South-West of the Mega escarpment are sealed by Quaternary lava flows erupted from vents at the Northeastern termination of the Hurri Hills (Supplementary Fig. 12b). A few minor normal faults affecting basal Hurri Hills lavas (see area indicated with arrow in Supplementary Fig. 12) suggest that very limited extensional deformation in the area may have occurred during the initial phases of building of this volcano.

The only published data on the Hurri Hills activity are the K/Ar ages by Brotzu et al.[10] and those reported in the geological map of the North Horr[23,24] (see also ref. 25). According to these ages and to the geological map, the present edifice is formed by a Late Pliocene basal lava pile, forming a large shield volcano, overlain by the products (lava flows and tephra

blankets) of a large number of Pleistocene monogenetic vents (mainly cinder cones) located on the shield surface.
The basal flood lavas of the edifice started emplacing at around 3 Ma, with a sample from the southwestern slope of the edifice giving a poorly constrained K/Ar age of 2.8±1.3 Ma (refs. 23,24; Supplementary Figure 7). This timing is consistent with these lavas overlying the Bulal basalts, which we dated at 3.6-3.7 Ma in the area of Dilo. Conversely, the lava flows and tephra products of the following monogenetic activity span quite a large time period, from an oldest age of 2.3±0.17 Ma (K/Ar age of ref. 10) up to a mid-Pleistocene age (K/Ar age of 0.54±0.10 Ma reported in refs. 23,24) for some products of summit vents.
As stated above, the lavas of the Hurri Hills shield seal the Ririba faults. Therefore, since the basal lavas of this shield, whose oldest reported age is 2.8 Ma, are only weakly affected by few minor normal faults at its northern termination and they are overlain by undeformed younger products with ages as old as 2.3 Ma, the end of rift activity likely occurred close to the Pliocene-Pleistocene boundary. Of course, the possibility exists that the lavas covering the faults may be even older than the poorly constrained age of 2.8 Ma (e.g., the ca. 3 Ma age reported in refs. 23,24), therefore further reducing the duration of faulting in the Ririba rift. Future work is needed to decrease the uncertainty in the timing of rift deactivation.

Supplementary Note 6: Analysis of the Mega escarpment

In order to provide additional constraints on the evolution and distribution of deformation in the area, we have analysed the morphostructural evidences for recent or current activity along the Mega escarpment, which represents the Eastern margin of the Turkana depression. The escarpment exposes high grade metamorphic rocks (mostly gneiss) of the Precambrian basement, locally covered by recent basaltic lava flows, scoria cones and maars of the Mega-Megado volcanic field.
The ~50 km long escarpments, separating the depression (elevations <900 m asl) from the Mega plateau (elevations >1400 m asl) and reaching a maximum height of >1200 m, is separated into two main segments by a fluvial valley (Supplementary Fig. 13). Apart from three ephemeral rivers that interrupt it, most of the escarpment is incised by small drainages whose channels rarely breach into the plateau.
The escarpment is strongly eroded so that it is possible just locally to recognize few triangular facets. To get a quantitative indication on the activity of the tectonic structure that generated the escarpment, we measured the mountain front sinuosity following ref. 26:

$$S = \frac{L_{mf}}{L}$$

where L_{mf} is the mountain-piedmont junction and L is the overall length of the mountain front. The resulting value of 1.7 is included in Class 2, interpreted as typical of a low tectonic activity[26].

At the bottom of the escarpment there is a pediment dipping to the SW and covered by many fans close to the mountain front. The fans appear to be inactive: the very few active channels are debris flow-like. Probably they occur during the main rainy season (April and May) when it rains ~53% of the scarce annual precipitation (~100 mm yr^{-1}; refs. 27,28). Although the fans are inactive, it is possible to recognize three different types, named F1, F2, F3 (Supplementary Fig. 13). F1 type, located in correspondence with the present three ephemeral rivers cutting across the Mega escarpment, presents very low slope (1-3°), is large in shape (~10-12 km wide) and could include more than one fan, some of which have the apex far from the mountain front in a telescopic pattern. The second type (F2) has a low slope (3-5°) and forms a sort of belt (~5 km wide) at the bottom of the Mega escarpment in correspondence with channels draining just the scarp. The relationship between F1 and F2 landforms indicates a contemporary deposition. On top of F2 lies the third type (F3), that include small (~1 km wide) and steep (5-8°) fans locally incised by active debris flow-like channels.

The configuration of the fans suggests that in the past, during a phase of wetter climate (likely the African Humid period, 15-5 ka), rivers draining the Mega Plateau were able to generate wide fans: their telescopic pattern is usually associated with rivers characterized by an erosion rate higher than the deformation rate at the mountain front[29,30,31], therefore supporting the interpretation based on the mountain front sinuosity. In the present semi-arid conditions, the steepness of the small fans (F3 type) at the bottom of the escarpment, as well as the active short channels, are more typical of a debris flow dynamic.

Notably, to the South of the study area, two very recent lava flows belonging to the volcanic field of Mega-Megado follow the channels along the escarpment from the high standing plateau down to the lowlands with no displacement.

According to these considerations, the fault that generated the Mega escarpment is inactive or has an activity so low that the surface processes easily overcome any possible displacement.

Supplementary Note 7: Numerical models

The complete evolution of rifting in the numerical models is illustrated in Supplementary Fig. 14. As explained in the main text, model results clearly illustrate the localization of deformation in the two offset rift valleys, which interact in correspondence to the transversal heterogeneity. During the initial phases of extension (12-24 km of bulk extension, roughly corresponding to 12-9 m.y. before present), deformation within the transversal domain is diffused over a wide region, in line with what is observed in previous modelling[3]. For increasing bulk extension (36 km of bulk extension, corresponding to 6 m.y. ago), the region affected by tectonic activity becomes narrower, focusing in the two overlapping tips of the propagating rift valleys (see labels T_E and T_K in Supplementary Fig. 14). In the latest stages of the model (60 km of bulk extension, corresponding to the present day), the two rift valleys directly connect, cutting away the southern tip of the Ethiopian rift valley (i.e., the Ririba rift in nature) from active deformation.

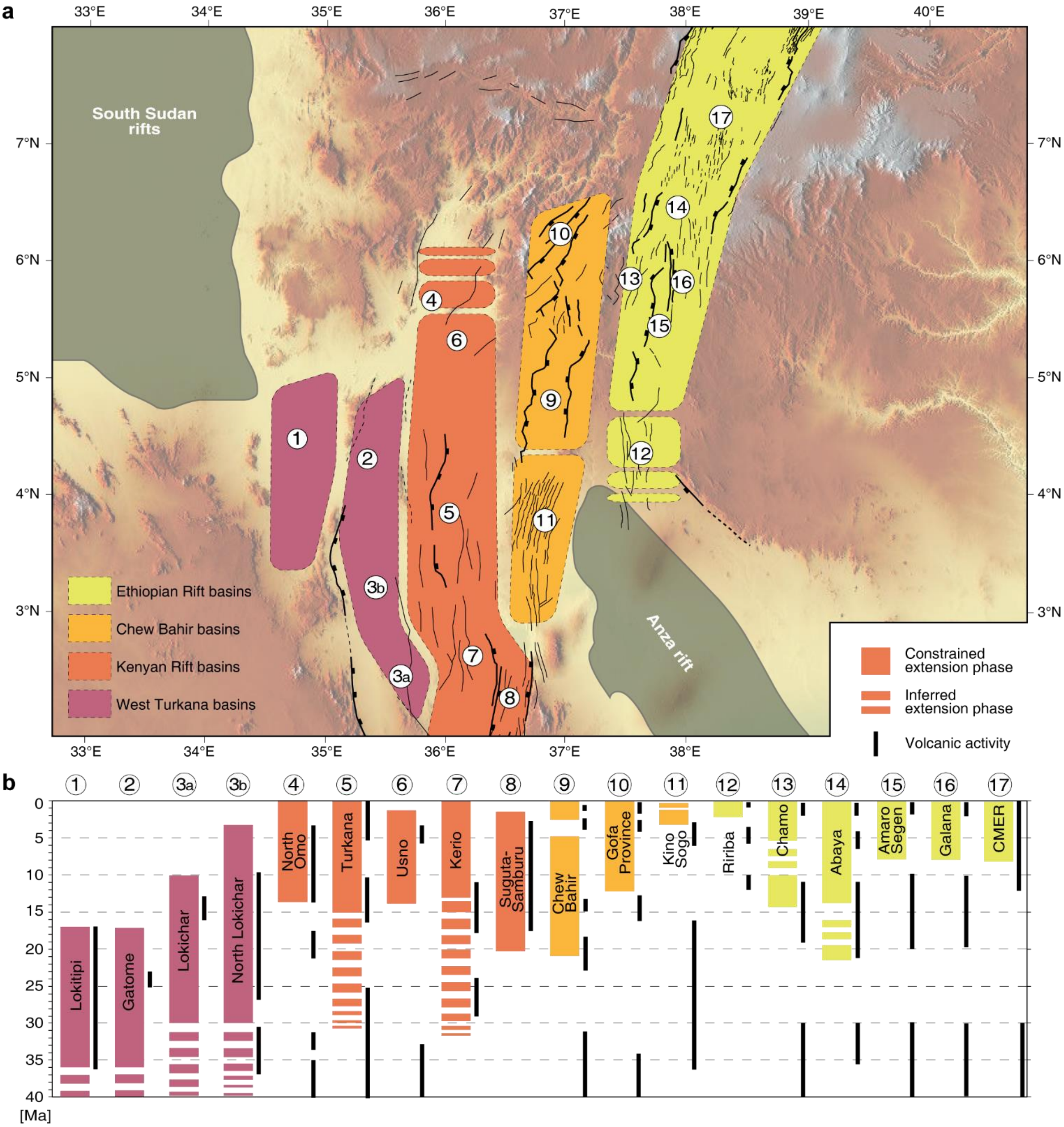

Supplementary Figure 1. a) Distribution of the main basins in the Turkana depression and surrounding areas, grouped in deformation domains (West Turkana, Kenyan rift, Chew Bahir, Ethiopian rift). b) Chronogram illustrating the spatial and temporal relationship of late Tertiary extension and volcanic activity (modified from ref. 9 and references therein, and integrated with refs. 10-15).

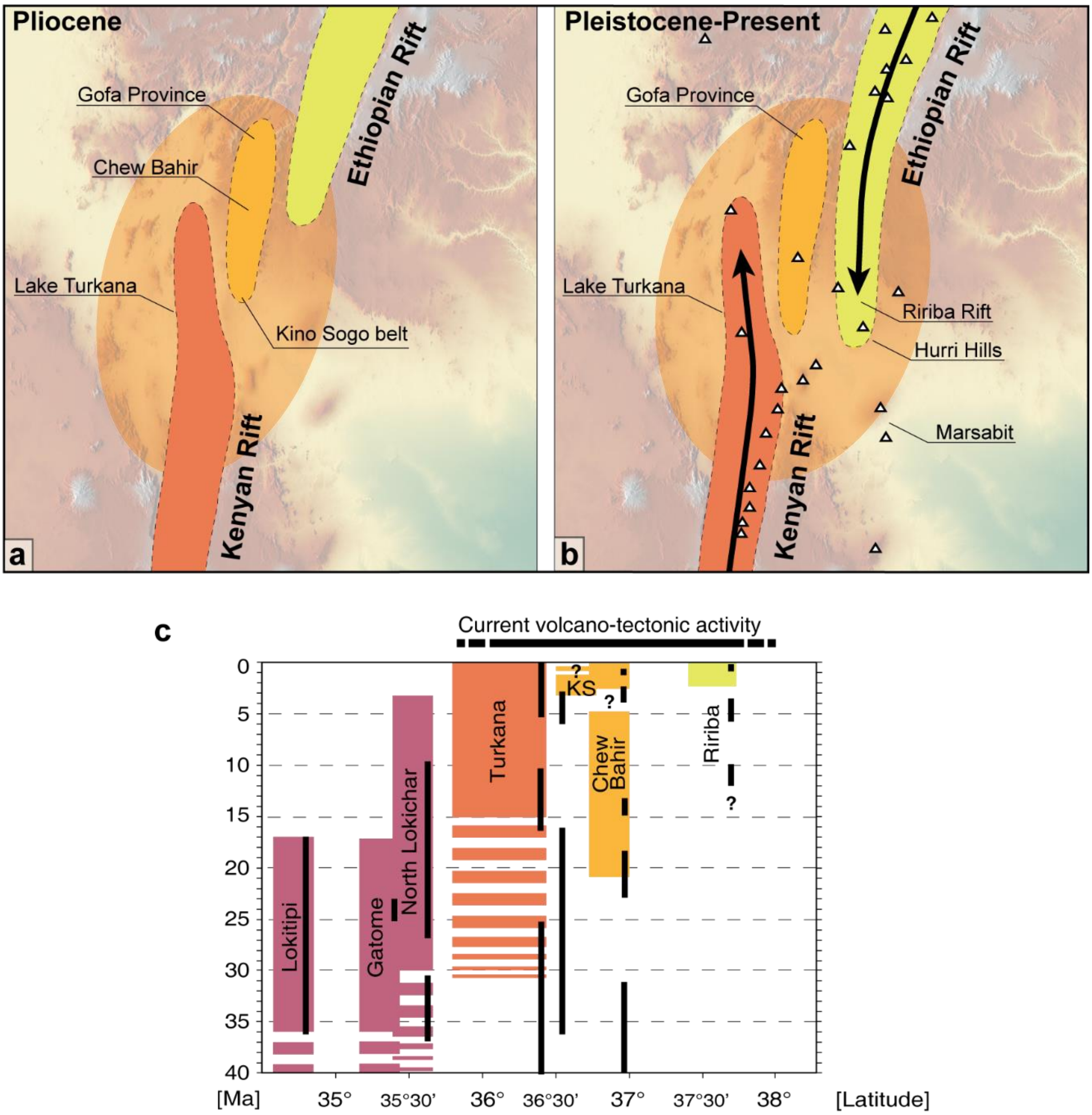

Supplementary Figure 2. Previous state of knowledge on the recent tectonic evolution of the Turkana depression prior to our study. a-b) Pliocene-recent schematic evolution of rifting. Coloured patterns delineate the main domains of tectonic and magmatic activity related to the Ethiopian and Kenyan rifts; the elliptical light orange pattern indicates the hypothesized extension of the region affected by deformation; black lines with arrows indicate the patterns of rift propagation[8]. c) Graph depicting the spatial-temporal relationship of recent extension in the axial portion of the Turkana depression (latitudes 3°N to 4°N), illustrated as in Supplementary Fig. 1. The distribution of the current volcano-tectonic activity shown by black bar is from ref. 2. KS: Kino Sogo. The location of the Lokitipi, Gatome and North Lokichar basins is illustrated in Supplementary Fig. 1.

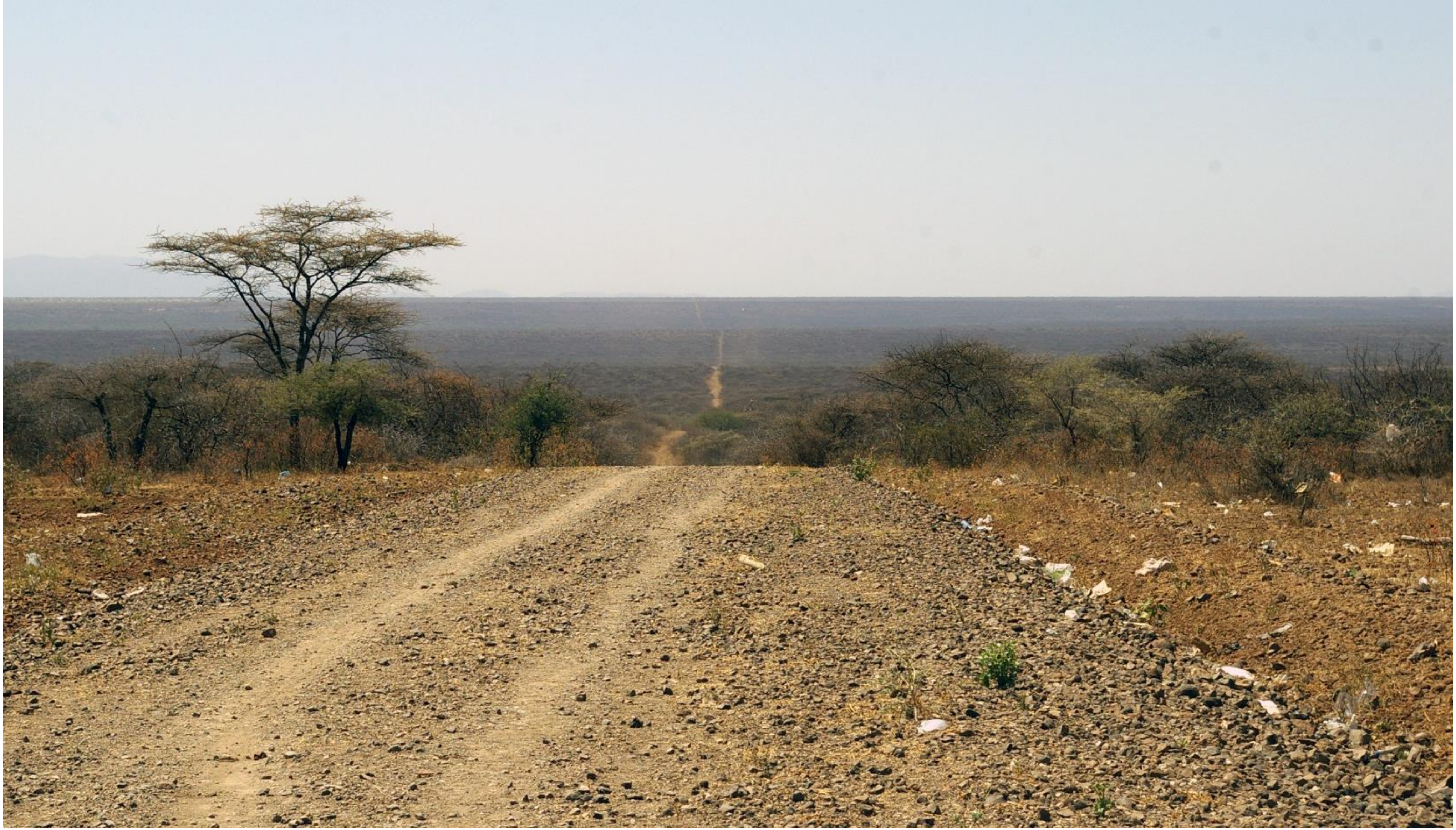

Supplementary Figure 3. Major normal faults at the western margin of the Ririba rift.

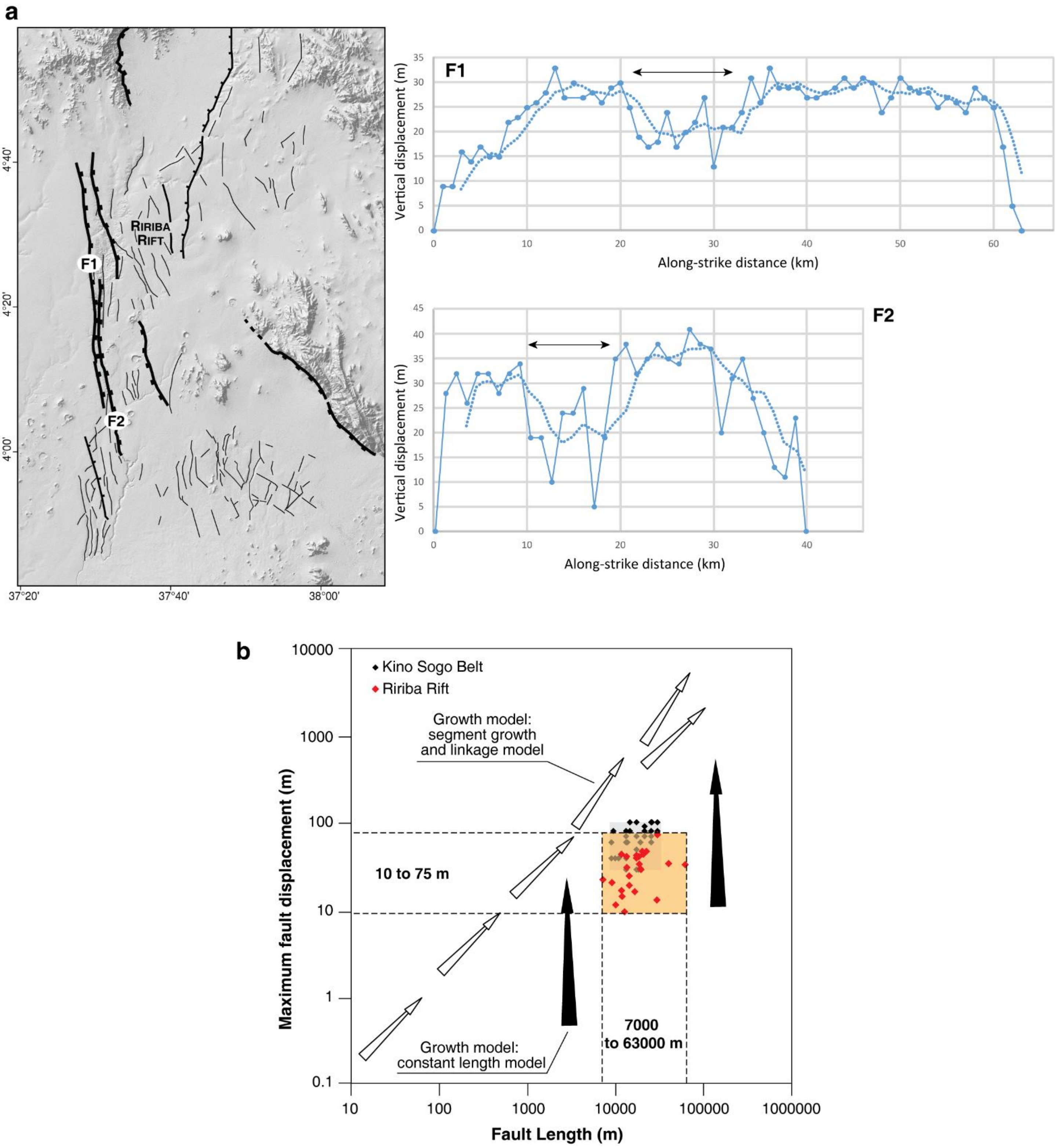

Supplementary Figure 4. a) Maximum displacement (D)/length (L) profiles (right panels; dotted line is D by using the moving window average) for major boundary faults (F1 and F2 in left panel) marking the western margin of the Ririba rift. Lines with double arrows indicate areas where the faults have been incised by small rivers, locally altering the D/L profiles. Note the flat top profiles, the low D-L values and the low-end gradients, which may indicate reactivation of old large basement faults. b) Log–log plot of maximum fault displacement versus length showing the 'under-displaced' nature of the rift-parallel normal faults in the Ririba rift, which are very similar to those measured in the nearby Kino Sogo belt[7]. White and black arrows indicate the two end-member growth models, namely the segment growth and linkage model and the constant length model[17]. Ririba fault data are in agreement with this latter growth model, in which fault lengths are established from an early stage and fault growth is dominated by displacement accumulation as observed in the nearby Kino Sogo belt[7].

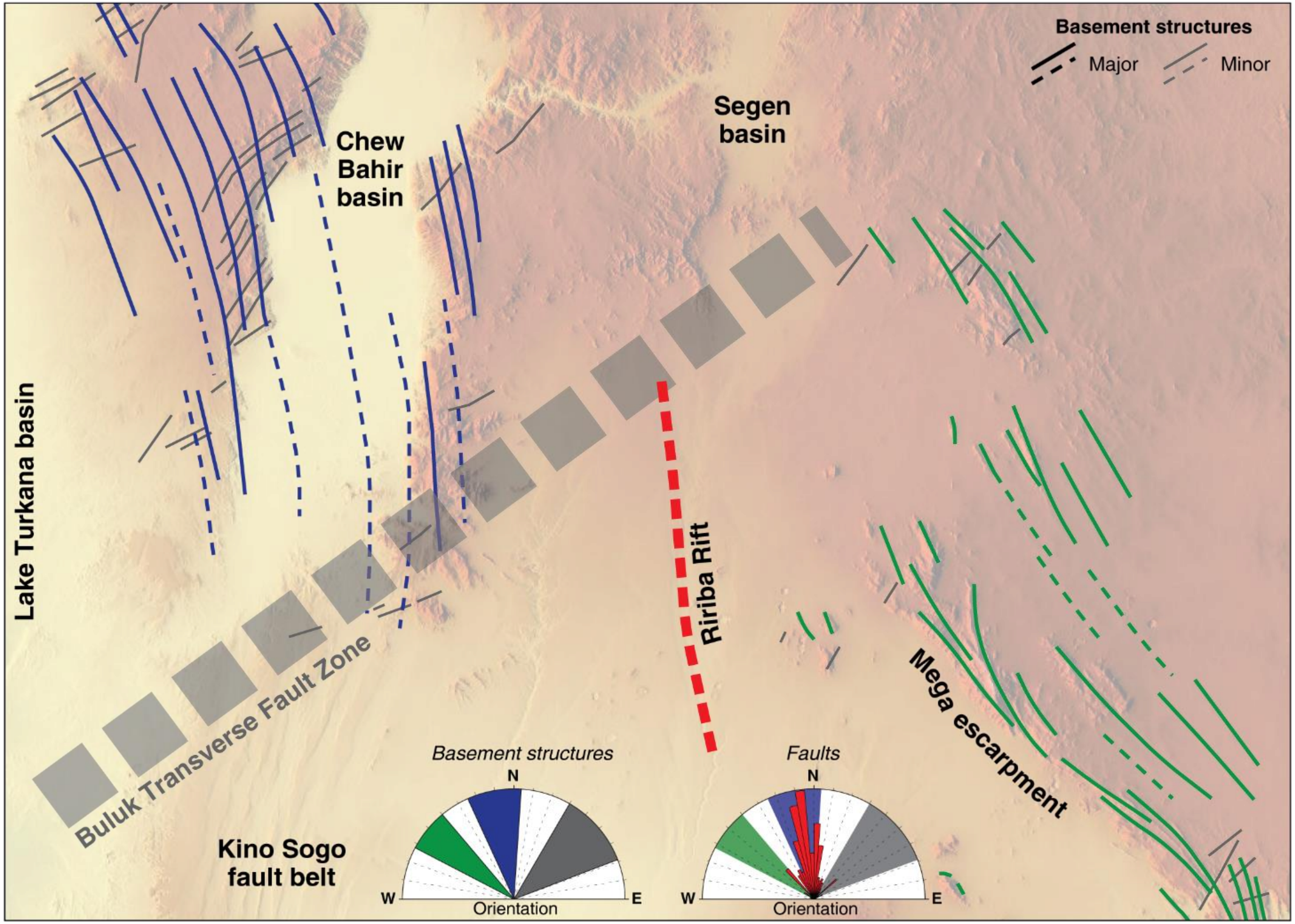

Supplementary Figure 5. Main basement fabrics in the area surrounding the Ririba rift (modified from ref. 7). Rose diagrams illustrate the distribution of basement structures and faults of the Ririba rift. Note that the majority of the faults are parallel to NNW-SSE fabrics (shown in blue); minor peaks in fault distribution correspond to the orientation of NW-SE and NE-SW basement fabrics (shown in green and dark grey, respectively).

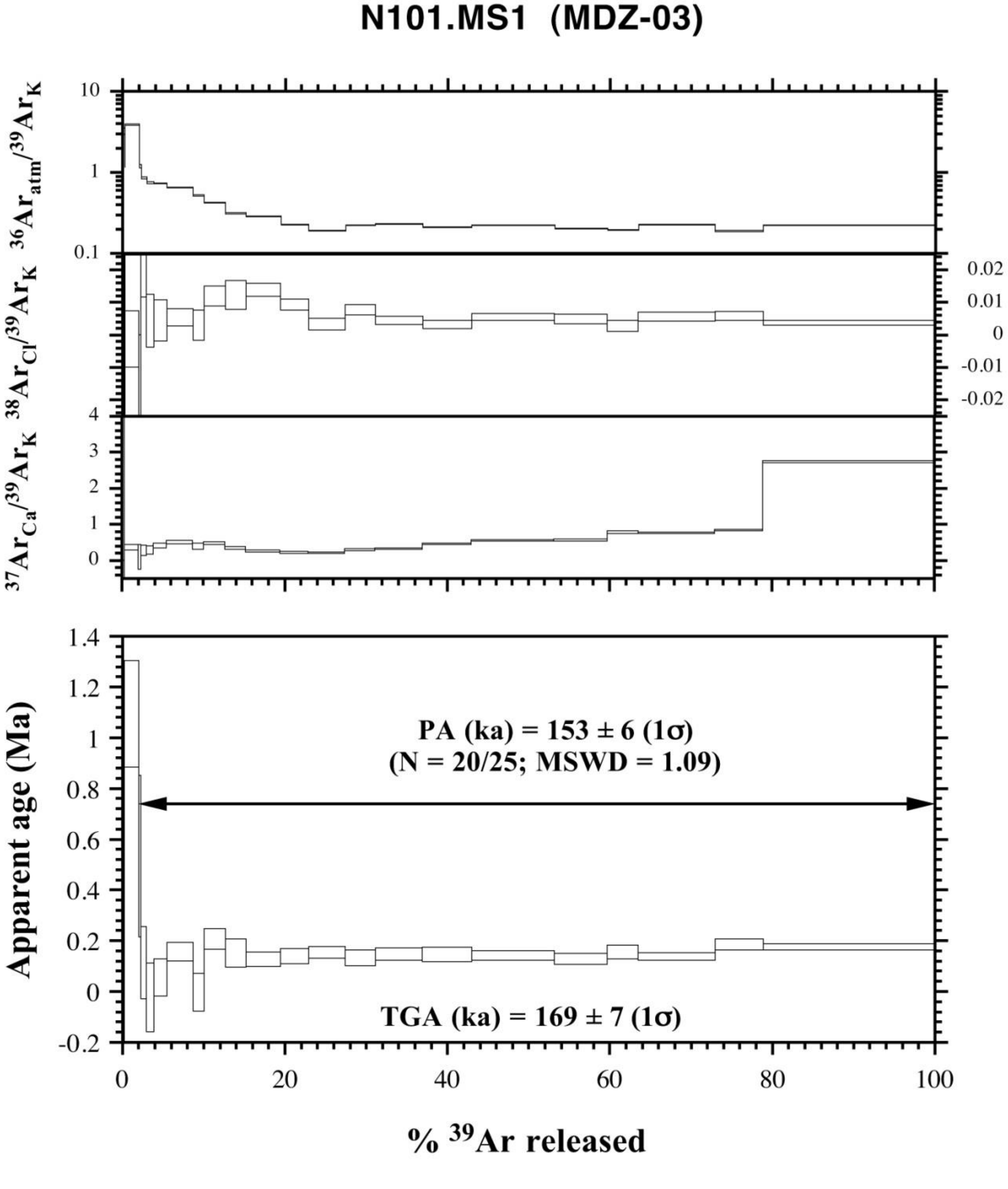

Supplementary Figure 6a-e. Ar/Ar data of samples MDZ03, 05, 10, 12, 13, reporting $^{37}Ar_{Ca}/^{39}Ar_{K}$, $^{38}Ar_{Cl}/^{39}Ar_{K}$, $^{36}Ar_{atm}/^{39}Ar_{K}$ ratios for the released gas (upper panel), Plateau Age (PA) with the respective estimated error (central panel) and Total Gas Age (TGA) with propagated error (lower panel).

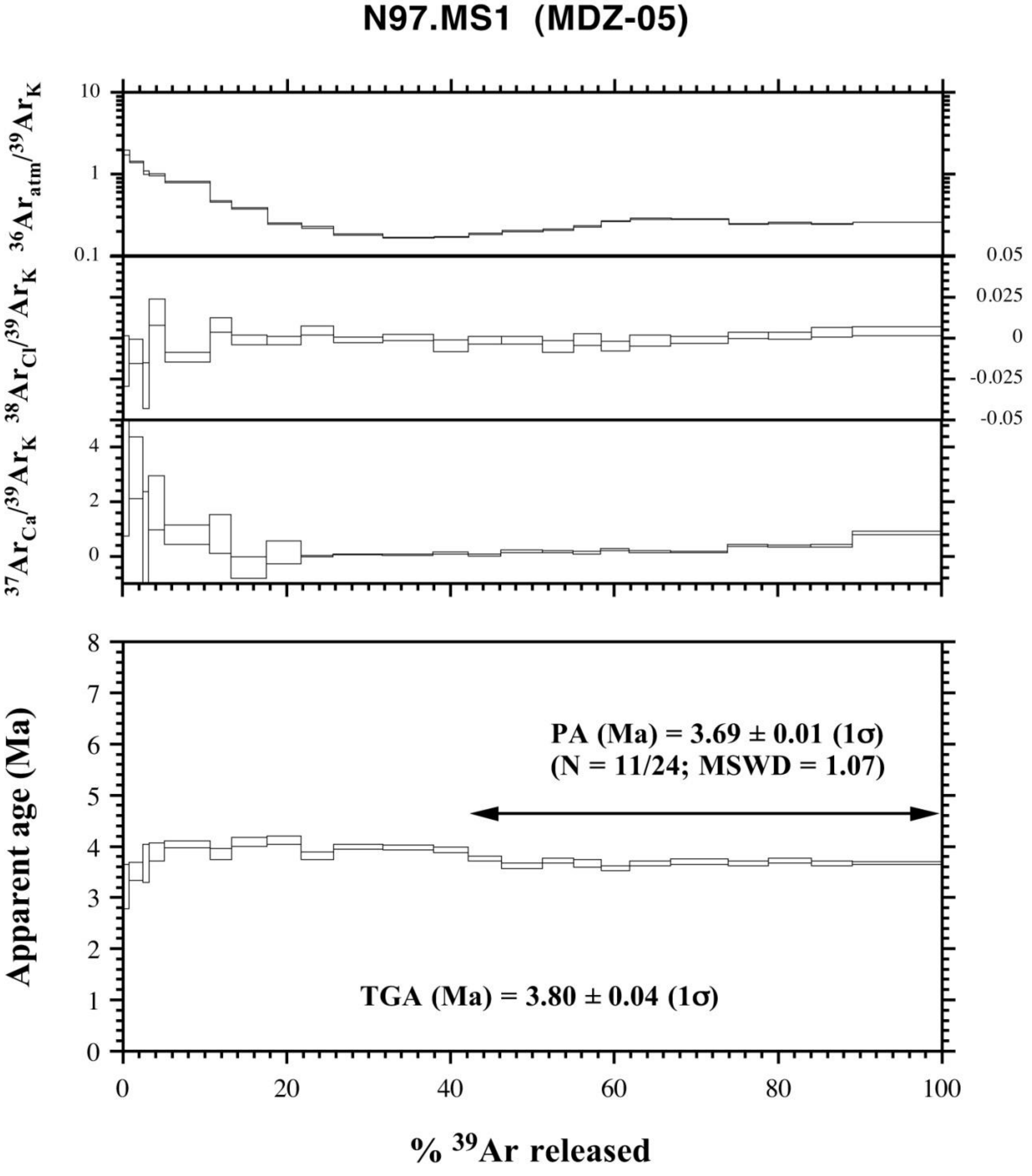

Supplementary Figure 6 continued.

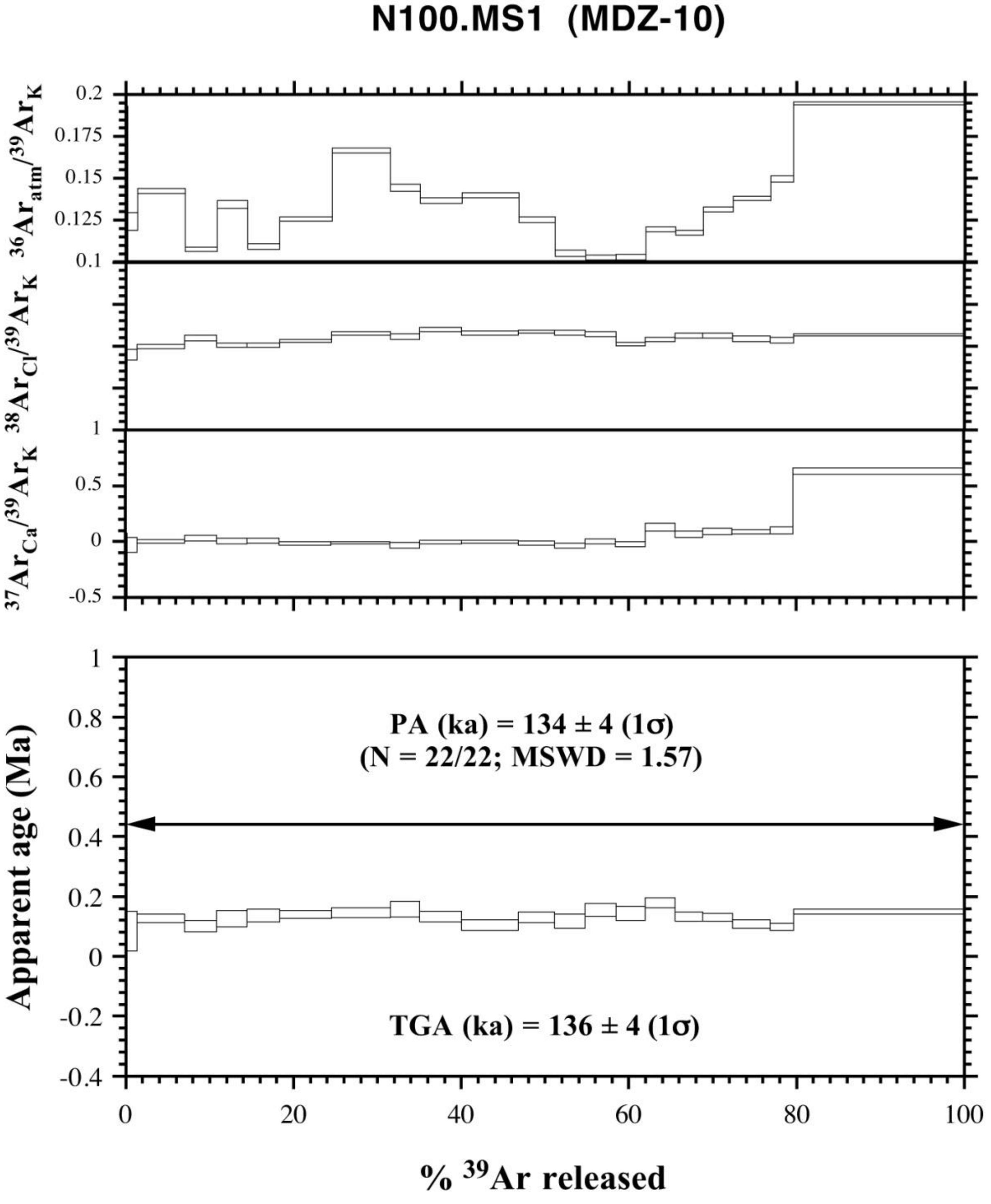

Supplementary Figure 6 continued.

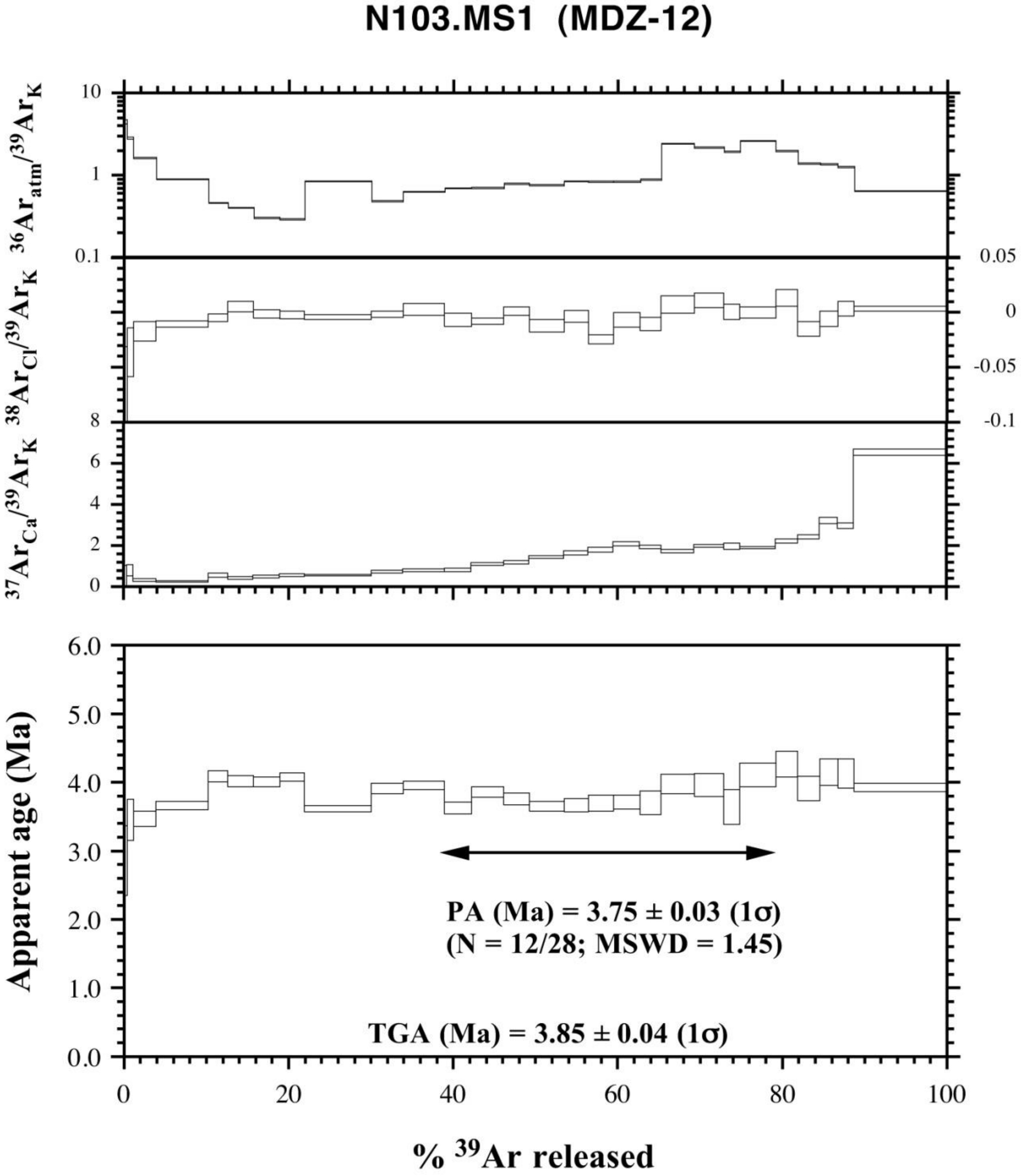

Supplementary Figure 6 continued.

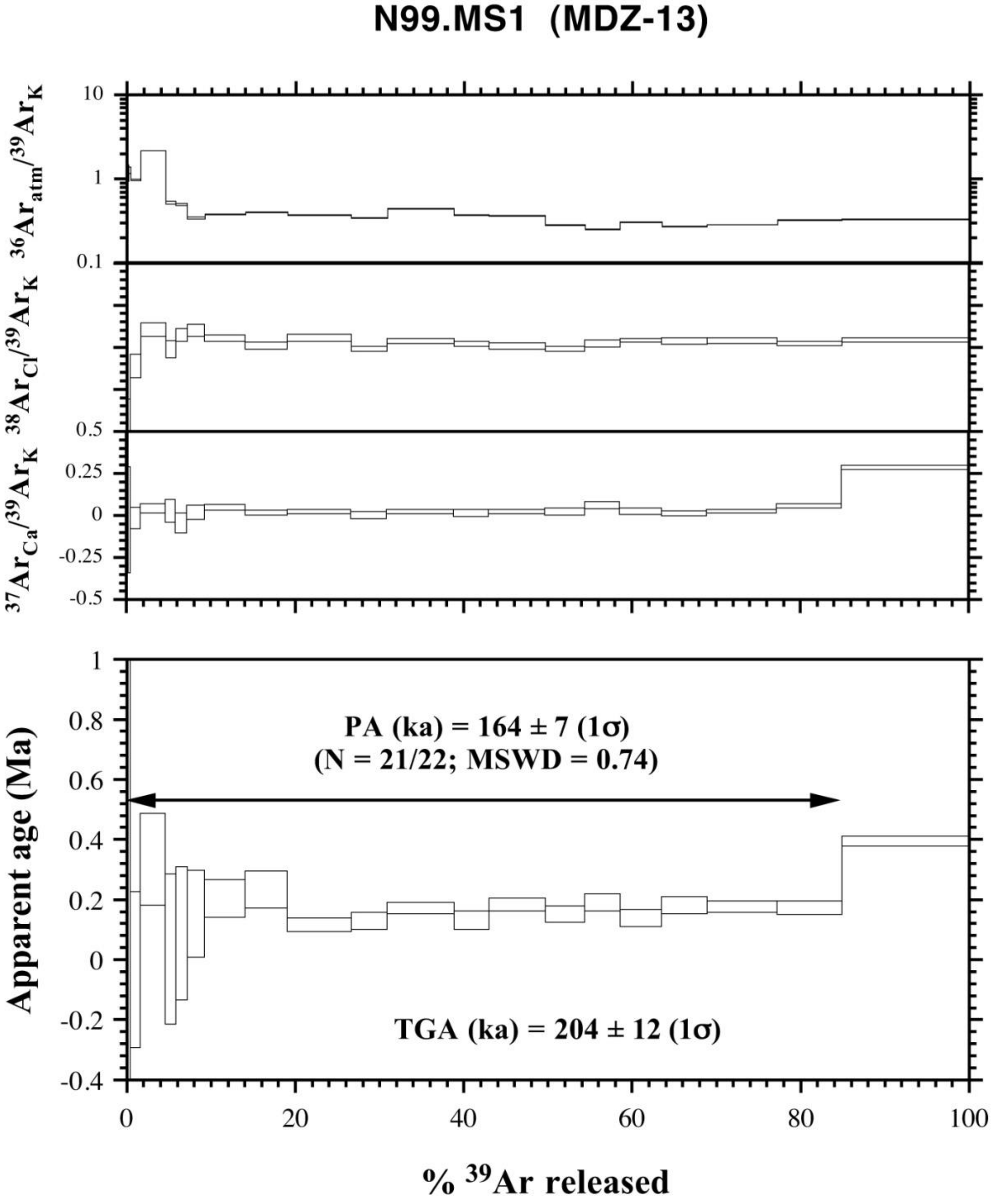

Supplementary Figure 6 continued.

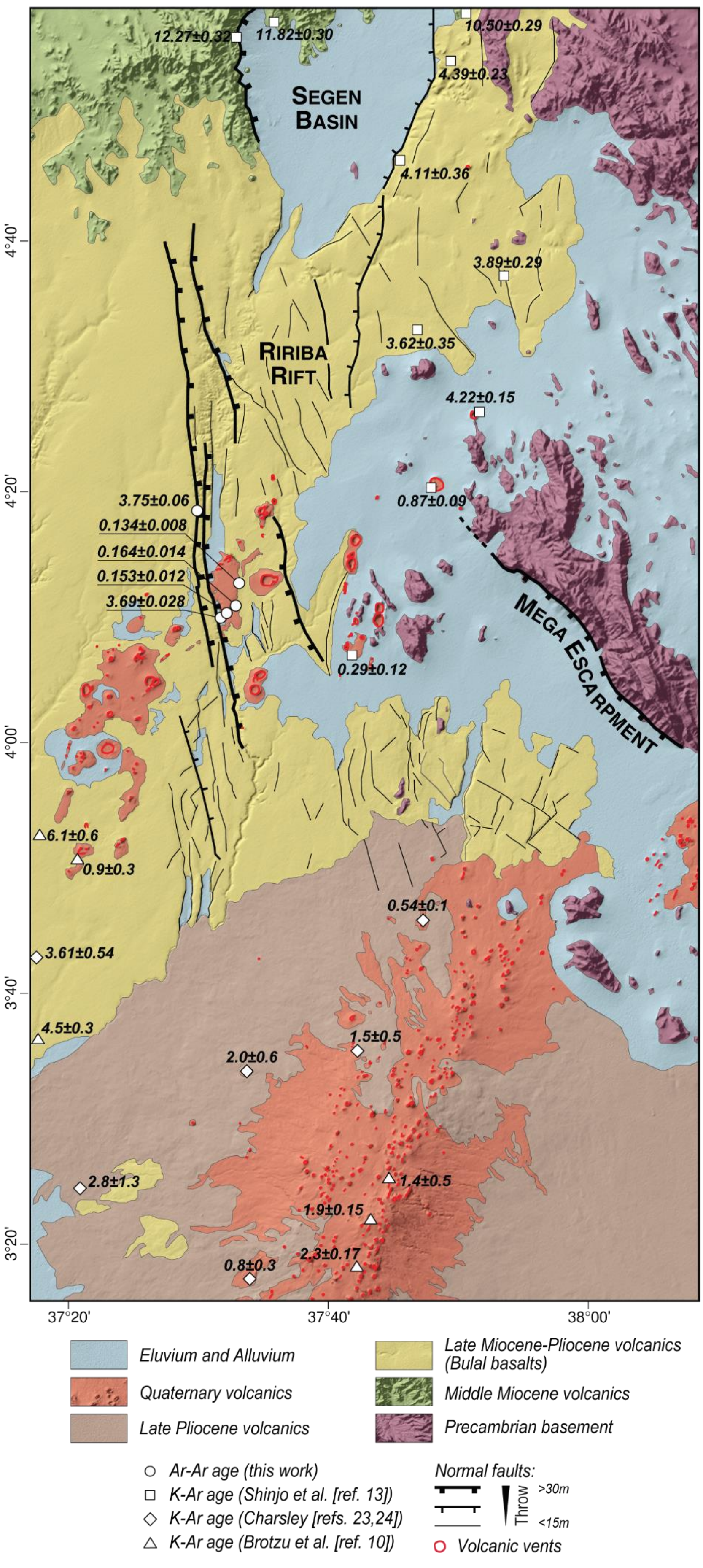

Supplementary Figure 7. Summary of geochronological data from the Ririba rift and surrounding regions, illustrated as in Figure 2.

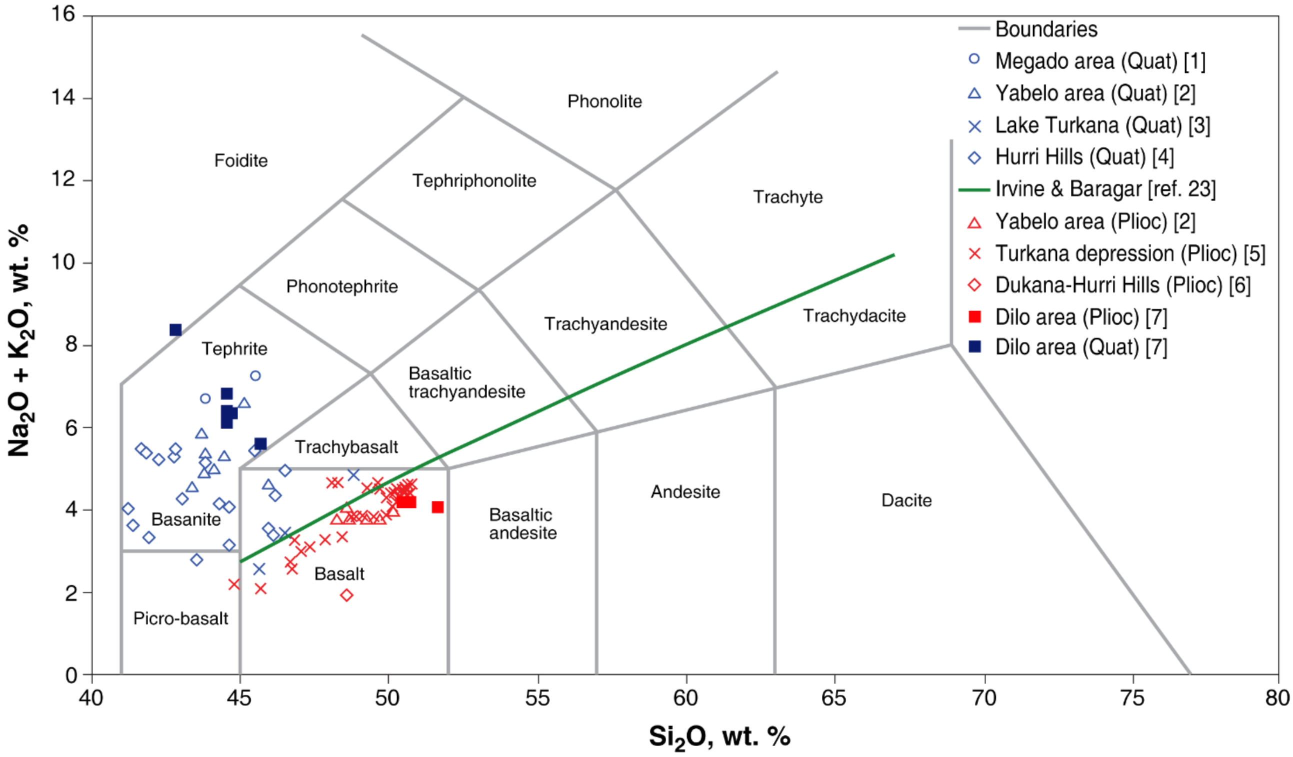

Supplementary Figure 8. Total Alkalies vs. SiO2 diagram (ref. 32) for selected rocks from the literature and data from this work. The green line separates the fields of alkaline and subalkaline rocks[32]. Pliocene rocks all plot in the subalkaline field, while Quaternary rocks have an alkaline affinity. Data from: [1] ref. 33; [2] ref. 13; [3] ref. 34; [4] ref. 35; [5] ref. 36; [6] ref. 10; [7] this paper.

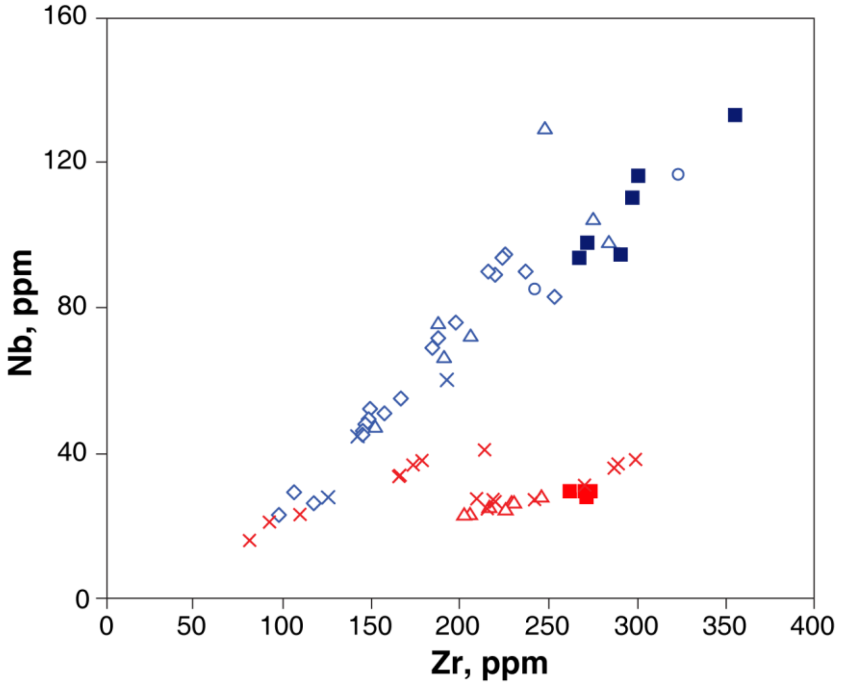

Supplementary Figure 9. Zr vs. Nb diagram showing the different trends related to the alkaline Quaternary and subalkaline Pliocene products from the Turkana-Ririba area. Symbols as in Supplementary Fig. 8.

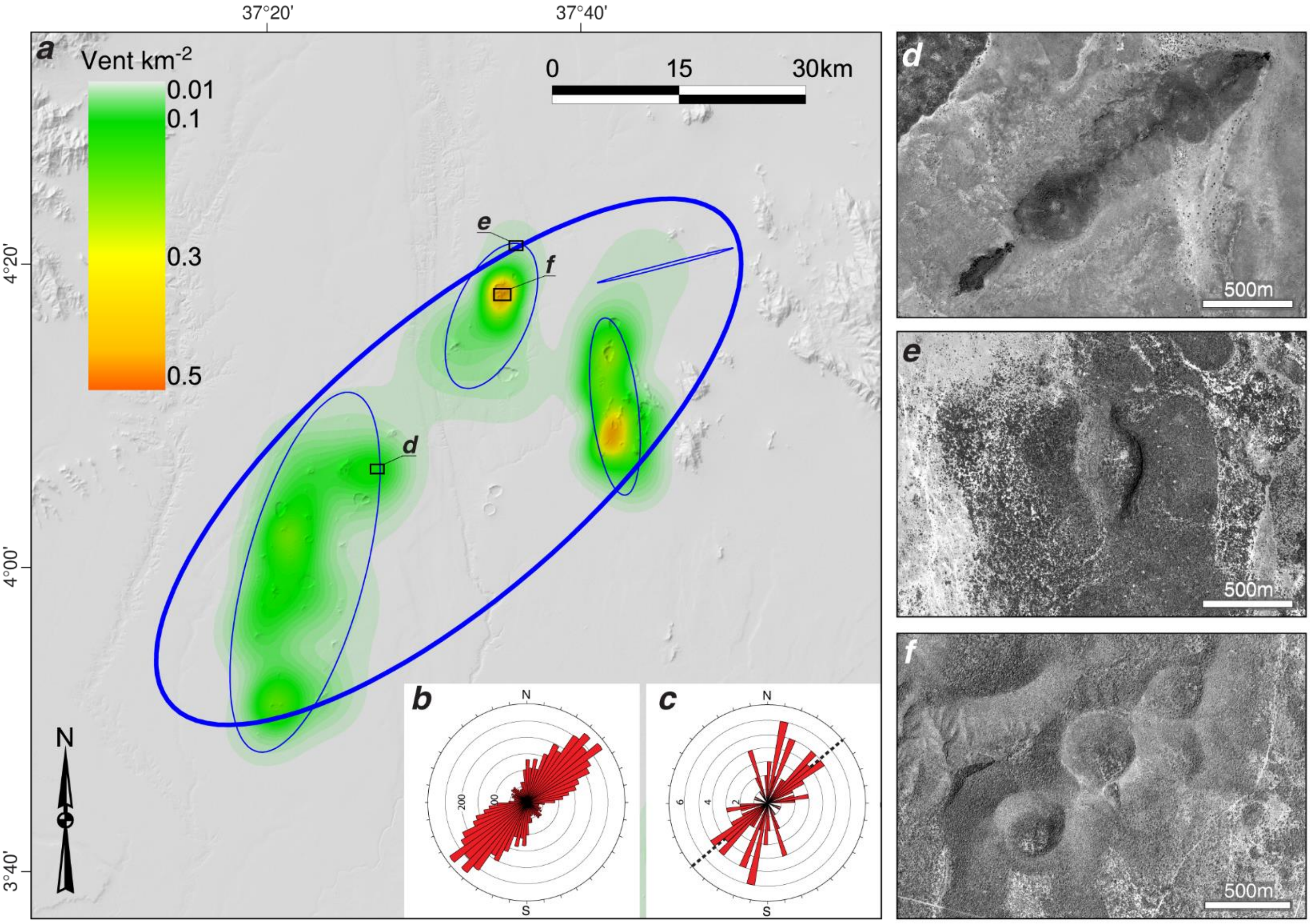

Supplementary Figure 10. a) Distribution of volcanic vents (illustrated as density of vents) in the Dilo-Dukana volcanic field from applying Equation (1). Ellipses are derived by applying to the original data sets (vent location in UTM coordinates -WGS84 datum) the Principal Component Analysis (PCA) as in ref. 37. The PCA describes the ellipse containing 95% of the data set and gives the azimuth of ellipse major axis. The same procedure has been applied also to the main vent clusters in the field. b) Rose diagram of the azimuth distribution of all the lines that connect the points in the data set (vents), the more elongated the volcanic field the more defined the main peaks in the rose diagram. c) Rose diagram of the azimuth distribution of structural features in the field such as eruptive fissures (d), vent elongation (e), vent alignments (f). The dashed black line indicates the overall elongation of the Dilo-Dukana volcanic field.

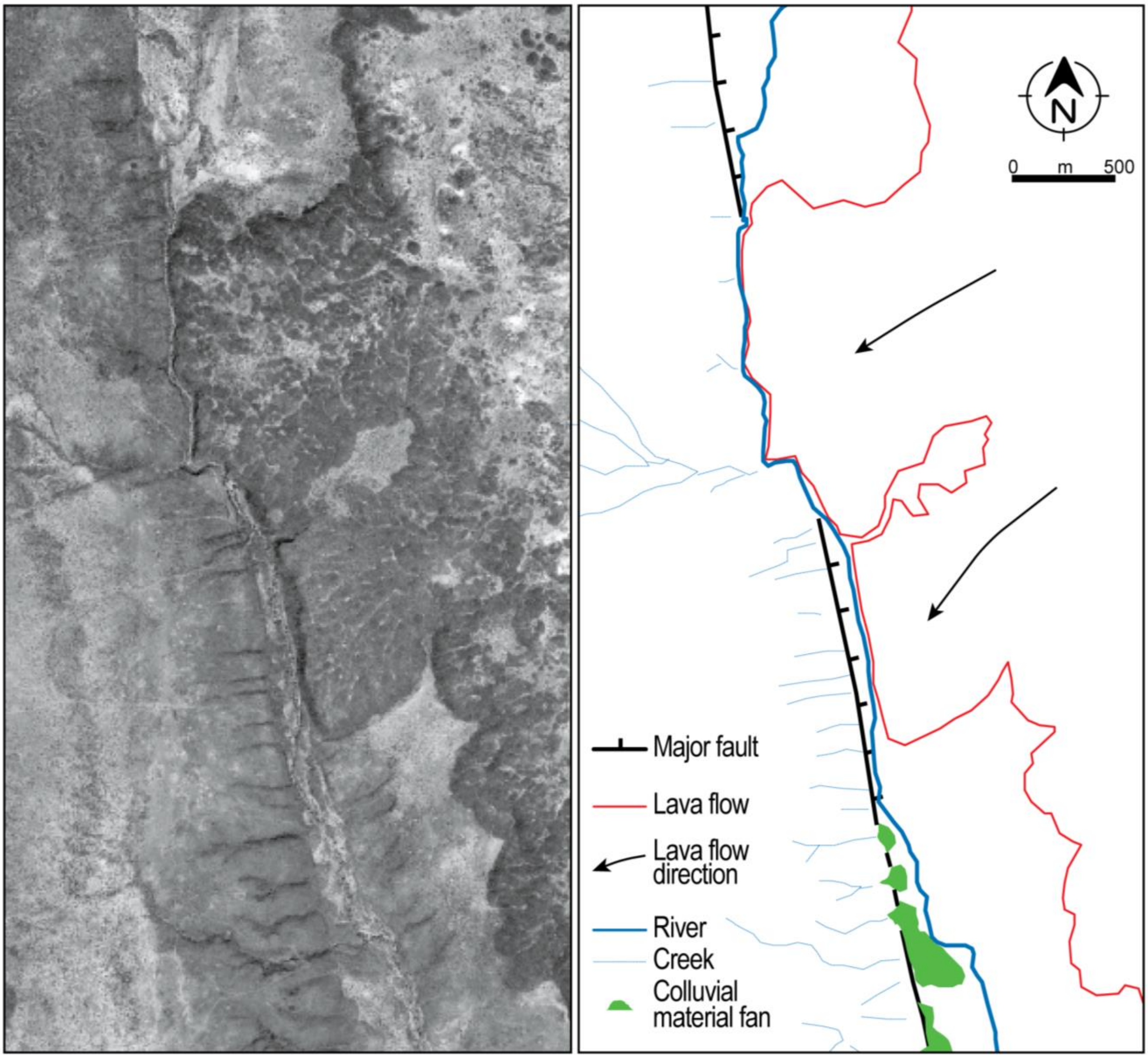

Supplementary Figure 11. Zoom of the central part of the Ririba rift (corresponding to the area labelled as A in Fig. 3) showing the large Late Pleistocene lava flow which partly filled the rift depression, forced the Ririba River to shift to the West and to incise a narrow valley partially eroding the fault footwall.

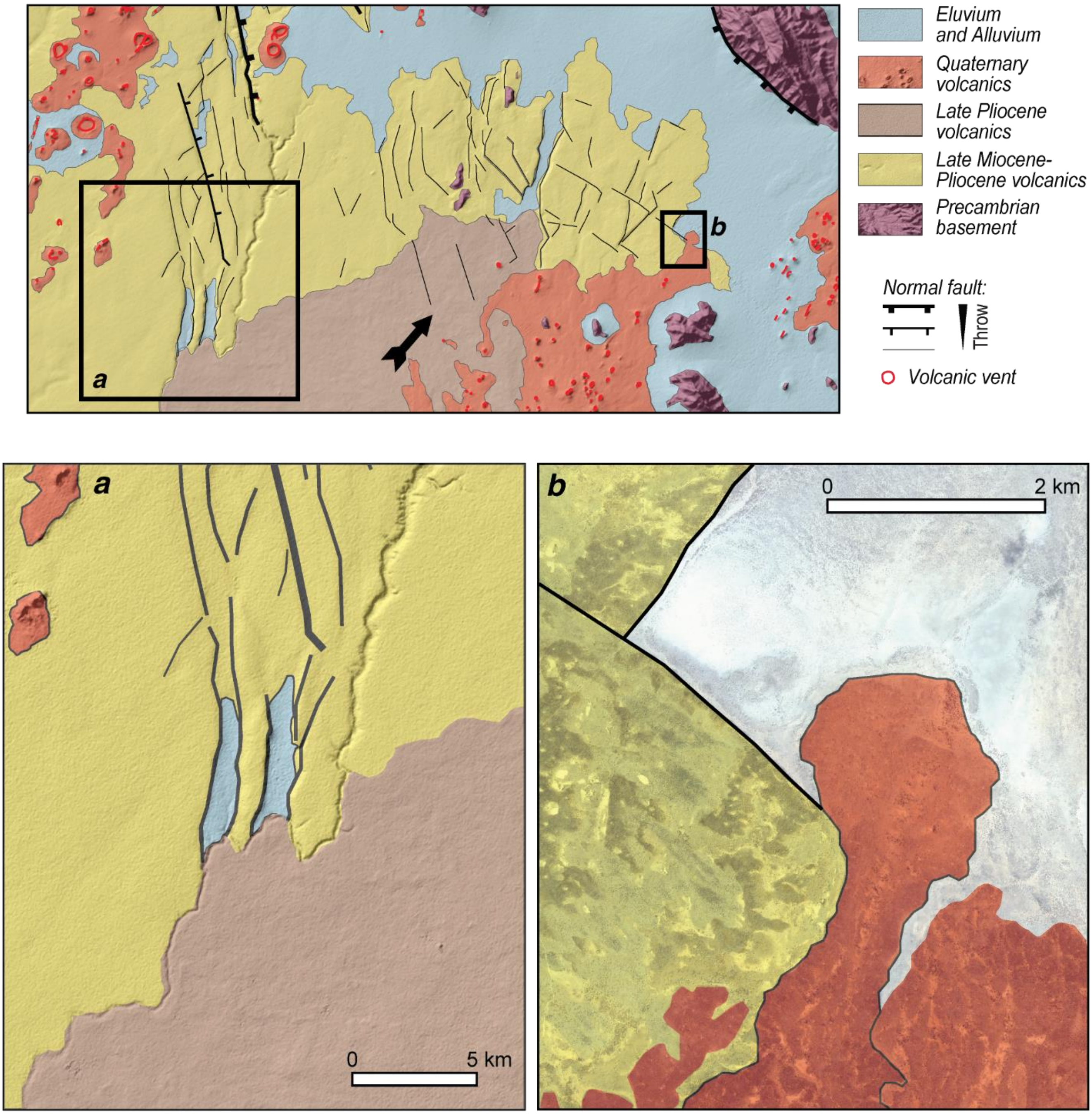

Supplementary Figure 12. Examples of rift-related faults affecting the Pliocene Bulal lavas sealed by recent basal flows of the Hurri Hills edifice at the southern termination of the Ririba rift (a) and South-West of the Mega escarpment (b). The arrows indicate the area where a few faults affect the basal lavas of the Hurri Hills edifice.

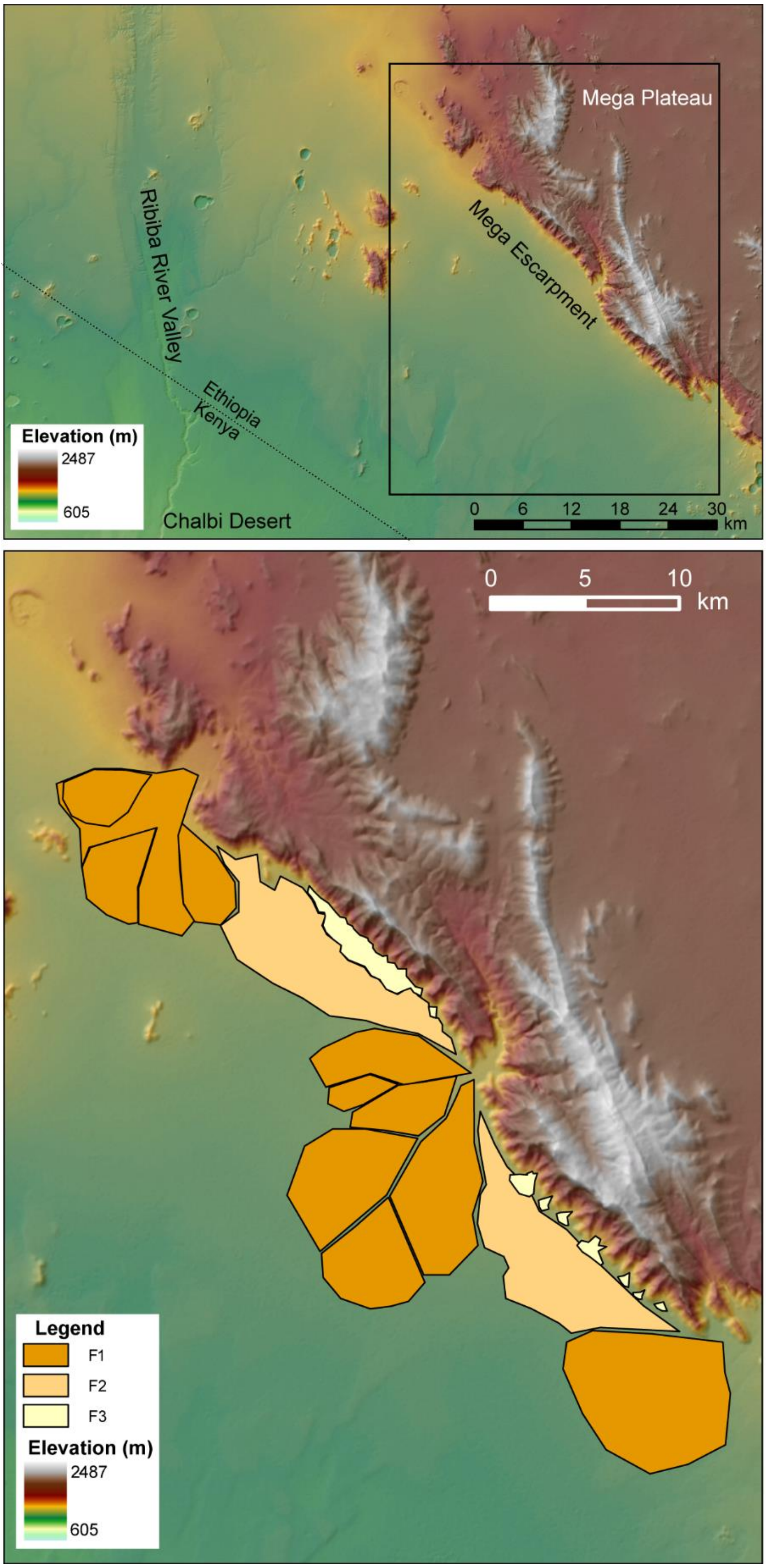

Supplementary Figure 13. SRTM (Nasa Shuttle Radar Topography Mission) digital elevation model of the Ririba rift and surrounding areas (upper panel), with close up of the Mega escarpment (lower panel) showing the three different types of fans labelled as F1, F2, F3.

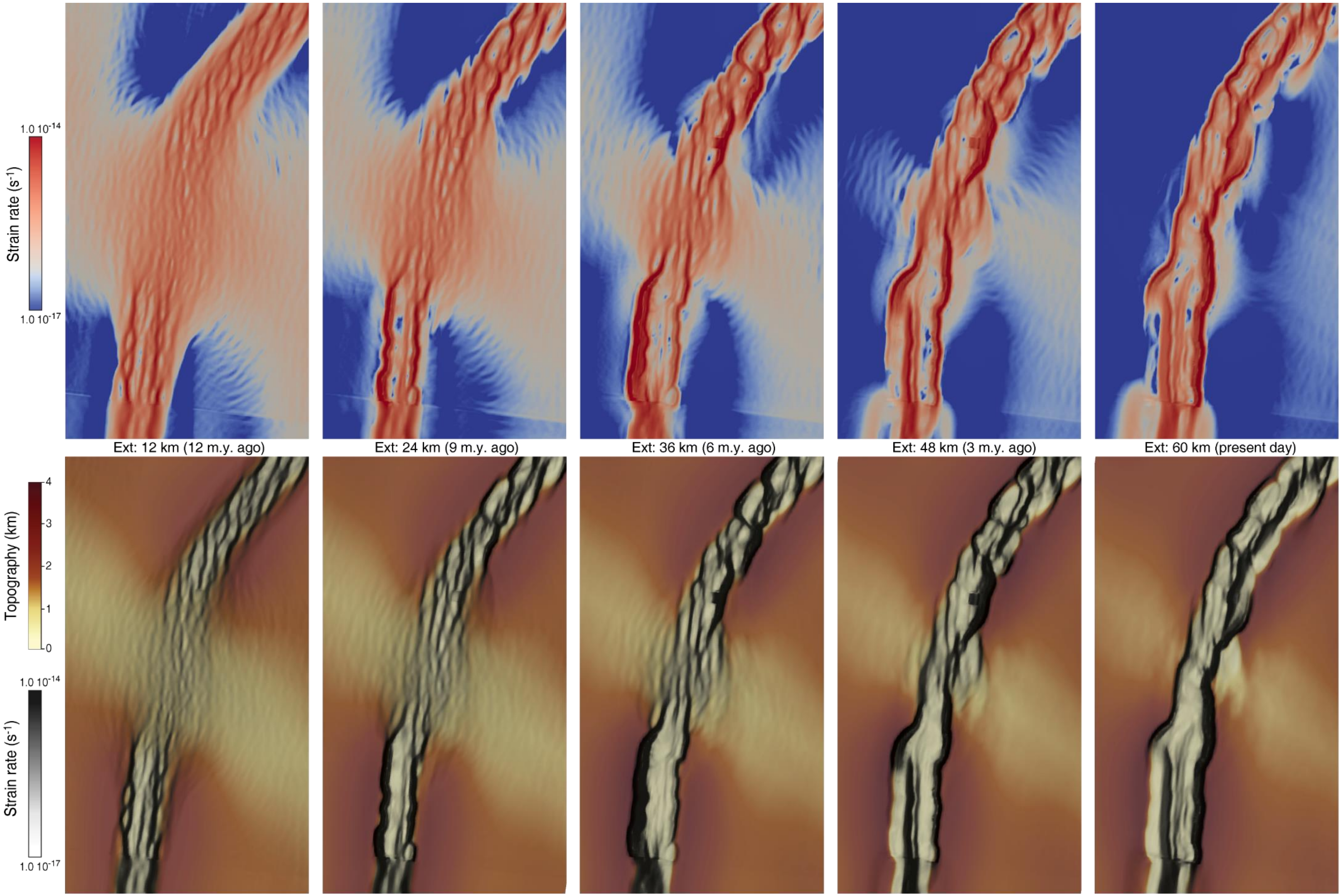

Supplementary Figure 14. Results of the numerical reference model. Evolution of the deformation pattern illustrated as second invariant of strain rate (top panels) and model surface topography (bottom panels, with semi-transparent strain rate field for reference) at different amounts of bulk extension. ER: Ethiopian rift; KR: Kenyan rift; T_E and T_K indicate the overlapping tips of the Ethiopian and Kenyan rift valleys, respectively; also reported are the regions approximately corresponding to the Gofa Province, Lake Turkana basin and the deactivated southern tip of the Ethiopian rift valley (i.e., the Ririba rift).

Supplementary Table 1. Results of Ar/Ar dating, reported with two different age estimates: the Total Gas Age (TGA) and the Plateau Age (PA). UpB and LwB indicates the Upper and lower cut-off values, respectively.

			Plateau age				MSWD/(N-1)				Total gas age			wt.%
Rock type	Phase	Name	(Ma)	±	2σ	LwB		UpB	n	N	(Ma)	±	2σ	K_2O
basalt	Gdm	MDZ-03	0.153	±	0.012	0.53	1.09	1.59	20	25	0.169	±	0.016	2.48
basalt	Gdm	MDZ-05	3.686	±	0.028	0.39	1.07	1.83	11	24	3.799	±	0.082	1.07
basalt	Gdm	MDZ-10	0.134	±	0.008	0.55	1.57	1.56	22	22	0.136	±	0.008	No data
basalt	Gdm	MDZ-12	3.749	±	0.060	0.42	1.45	1.79	12	28	3.850	±	0.088	1.02
basalt	Gdm	MDZ-13	0.164	±	0.014	0.54	0.74	1.57	21	22	0.204	±	0.024	1.83

Supplementary Table 2. Whole-rock geochemical analyses for major and trace element composition of representative samples.

		MDZ03	MDZ04	MDZ05	MDZ06	MDZ09	MDZ11	MDZ12	MDZ13	MDZ15
**Latitude (N)**		04°10'25	04°10'25	04°10'04	04°09'19	04°12'28	04°12'31	04°18'25	04°11'14	04°14'42
**Longitude (E)**		37°32'09	37°32'14	37°31'37	37°27'49	37°35'06	37°33'10	37°29'45	37°32'58	37°42'06
**Rock type***		Nepheline basanite	Tholeiitic basalt	Tholeiitic basalt	Nepheline basanite	Nepheline basanite	Nepheline basanite	Tholeiitic basalt	Nepheline basanite	Nepheline basanite
**SiO2**	wt.%	41.4	48.4	49.1	43.9	44.5	43	49.2	43.4	43.6
**Al2O3**	wt.%	13.85	13.55	13.8	14.95	12.3	12.15	12.95	12.7	13
**Fe2O3**	wt.%	15.95	14.65	14.4	13.15	12.9	13.9	14.2	13.95	13.85
**CaO**	wt.%	9	8.19	8.38	9.43	9.41	8.74	8.27	9.45	9
**MgO**	wt.%	5.85	4.52	4.38	7.97	11.15	10.3	4.11	9.27	10.25
**Na2O**	wt.%	5.6	2.95	2.97	4.68	3.79	4.26	2.84	4.32	4.29
**K2O**	wt.%	2.48	1.08	1.07	2.04	1.63	1.94	1.02	1.83	1.72
**TiO2**	wt.%	2.64	3.33	3.4	2.59	1.89	2.24	3.39	2.15	2.32
**MnO**	wt.%	0.29	0.22	0.21	0.24	0.24	0.26	0.21	0.28	0.24
**P2O5**	wt.%	1.09	0.47	0.49	0.77	0.68	0.84	0.48	0.86	0.8
**LOI**	wt.%	-0.22	0.94	1.33	0.27	-0.09	0.57	1.65	-0.01	-0.26
**Total**	wt.%	98.18	98.35	99.6	100.22	98.67	98.46	98.4	98.46	99.05
**Co**	ppm	40	39	39	42	55	51	41	45	53
**Cu**	ppm	46	61	61	66	66	51	68	59	62
**Li**	ppm	10	10	10	10	10	10	10	10	10
**Ni**	ppm	60	22	23	148	345	325	23	254	305
**Pb**	ppm	9	5	7	2	12	5	10	10	bdl
**Sc**	ppm	13	29	29	22	22	17	30	20	20
**Zn**	ppm	125	138	141	105	104	115	143	111	112
**Ba**	ppm	1025	303	378	827	677	893	418	922	720
**Ce**	ppm	165	62.9	62.5	152	129.5	145.5	63.9	159	136
**Cr**	ppm	60	10	bdl	220	680	410	10	340	430
**Cs**	ppm	1.05	0.18	0.22	0.48	0.7	0.81	0.37	0.82	0.64
**Dy**	ppm	8	7.53	7.75	7.47	6.17	6.81	7.96	7.06	6.83
**Er**	ppm	3.73	4.32	3.96	3.35	3.41	3.26	4.37	3.81	3.24
**Eu**	ppm	3.81	2.84	2.75	3.48	3.03	3.25	2.83	3.43	3.3
**Ga**	ppm	21.3	25	24.6	21	17.1	18.3	23.8	18.3	18.5
**Gd**	ppm	10.25	9.01	8.31	9.32	8.15	8.6	8.45	9.37	8.72
**Hf**	ppm	8.2	6.3	6.6	6.7	6.4	6.5	6.4	6.3	5.9
**Ho**	ppm	1.35	1.5	1.48	1.33	1.27	1.3	1.5	1.36	1.27
**La**	ppm	92.6	29.6	29.4	91.2	71	80.1	27.1	86.9	77.1
**Lu**	ppm	0.44	0.46	0.52	0.46	0.39	0.4	0.48	0.44	0.4
**Nb**	ppm	133	29.4	29.3	94.7	94.1	111	29.6	116	98.1
**Nd**	ppm	70.6	37.3	36.9	60.8	54.6	62.7	37.9	66.7	57.7
**Pr**	ppm	18	8.2	8.08	16.2	14.05	16.2	8.41	17.25	15.15
**Rb**	ppm	92.3	19.1	18.8	56.1	58.8	68.1	19.8	68.4	57.2
**Sm**	ppm	12.35	8.62	8.65	11.15	8.73	10.7	8.35	11.7	9.73
**Sr**	ppm	1190	360	357	1145	886	994	366	1035	1005
**Ta**	ppm	6.3	2	1.9	4.8	6.3	5.4	2.2	4.7	5.2
**Tb**	ppm	1.44	1.32	1.26	1.39	1.03	1.24	1.33	1.28	1.16
**Th**	ppm	12.3	2.9	2.76	9.97	10.45	11.25	2.97	12.25	10.05
**Tm**	ppm	0.53	0.58	0.55	0.49	0.47	0.42	0.58	0.49	0.4
**U**	ppm	2.72	0.83	0.74	2.38	2.29	2.5	0.6	2.59	2.2
**V**	ppm	134	428	423	191	167	155	423	172	184
**Y**	ppm	37.5	40.5	40.9	38.8	31.6	32.6	40.6	37.1	30.9
**Yb**	ppm	3.28	4.02	3.94	3.5	3.02	2.76	3.6	3.33	2.98
**Zr**	ppm	354	273	271	291	266	297	262	300	271

*classification according to Le Maitre et al.[38]

Supplementary Table 3. Analyses of standard samples and reference values.

		AMIS0304	*Ref. value*		GRE-3	*Ref. value*		SY-4	*Ref. value*		OGGeo08	*Ref. value*	
		Measured	*Median*	*± stdev*	*Measured*	*Median*	*± stdev*	Measured	*Median*	*± stdev*	Measured	*Median*	*± stdev*
**SiO2**	wt.%	12.05	*12.33*	*0.21*	3.33	*3.27*	*0.08*	50.5	*49.9*	*0.6*			
**Al2O3**	wt.%	1.57	*1.52*	*0.05*	0.93	*0.94*	*0.04*	20.7	*20.7*	*0.3*			
**Fe2O3**	wt.%	21.3	*21.0*	*0.3*	50.9	*50.5*	*0.6*	6.22	*6.21*	*0.13*			
**CaO**	wt.%	28.6	*28.5*	*0.4*	18.35	*18.35*	*0.27*	8.10	*8.05*	*0.16*			
**MgO**	wt.%	2.89	*2.87*	*0.08*	0.16	*0.14*	*0.02*	0.51	*0.54*	*0.03*			
**Na2O**	wt.%	0.09	*0.09*	*0.02*	0.04	*0.03*	*0.01*	7.06	*7.10*	*0.15*			
**K2O**	wt.%	0.27	*0.28*	*0.02*	0.04	*0.03*	*0.01*	1.64	*1.66*	*0.05*			
**Cr2O3**	wt.%	0.01	*0.02*	*0.01*	0.01	*0.02*	*0.01*						
**TiO2**	wt.%	1.82	*1.80*	*0.06*	1.63	*1.63*	*0.05*	0.30	*0.29*	*0.02*			
**MnO**	wt.%	0.46	*0.46*	*0.03*	1.71	*1.65*	*0.05*	0.11	*0.11*	*0.01*			
**P2O5**	wt.%	18.55	*18.35*	*0.27*	15.35	*15.25*	*0.25*	0.12	*0.13*	*0.02*			
**Co**	ppm										98	*97*	*5,5*
**Cu**	ppm										8350	*8390*	*295*
**Li**	ppm										30	*30*	*10*
**Ni**	ppm										8930	*8885*	*442,5*
**Pb**	ppm										7230	*7240*	*365*
**Sc**	ppm										10	*10,5*	*1,25*
**Zn**	ppm										7250	*7225*	*362,5*
**Ba**	ppm	2660	*2600*	*130*	6900	*6975*	*348*	352	*341*	*17*			
**Ce**	ppm	8150	*8090*	*405*	4340	*4355*	*218*	124	*122*	*6*			
**Cr**	ppm	90	*100*	*10*	40	*50*	*10*						
**Cs**	ppm	0.36	*0.40*	*0.03*	0.08	*0.11*	*0.01*	1.42	*1.50*	*0.08*			
**Dy**	ppm	133.0	*132.3*	*6.6*	86.2	*92.3*	*4.6*	19.8	*18.2*	*0.9*			
**Er**	ppm	34.2	*34.0*	*1.7*	26.8	*28.9*	*1.5*	15.2	*14.2*	*0.7*			
**Eu**	ppm	149.0	*150.0*	*7.5*	73.8	*75.3*	*3.8*	2.0	*2.0*	*0.1*			
**Ga**	ppm	49.2	*53.3*	*2.7*	37.8	*41.8*	*2.1*	38.3	*35.0*	*1.8*			
**Gd**	ppm	341.0	*343.0*	*17.0*	173.5	*191.0*	*9.5*	15.1	*14.0*	*0.7*			
**Hf**	ppm	27.8	*28.0*	*1.5*	18.7	*19.8*	*1.1*	12.4	*11.1*	*0.7*			
**Ho**	ppm	17.15	*18.00*	*0.90*	12.45	*13.53*	*0.69*	4.37	*4.30*	*0.22*			
**La**	ppm	3550	*3610*	*180*	2230	*2225*	*113*	59.9	*58.0*	*3.2*			
**Lu**	ppm	1.99	*2.05*	*0.11*	1.62	*1.81*	*0.10*	2.03	*2.10*	*0.11*			
**Nb**	ppm							13.6	*13.0*	*0.8*			
**Nd**	ppm	4260	*4010*	*200*	1845	*1835*	*93*	62.6	*57.0*	*2.9*			
**Pr**	ppm				477	*497*	*25*	15.75	*15.00*	*0.78*			
**Rb**	ppm	10.6	*10.6*	*0.6*	0.9	*0.7*	*0.2*	55.1	*55.0*	*2.9*			
**Sm**	ppm	605	*604*	*30*	267	*279*	*14*	13.40	*12.70*	*0.65*			
**Sr**	ppm	3450	*3400*	*170*	1985	*2093*	*104*	1290	*1190*	*60*			
**Ta**	ppm	11.8	*12.5*	*0.7*	145.5	*161.8*	*8.1*	0.8	*0.9*	*0.1*			
**Tb**	ppm	32.7	*34.3*	*1.7*	19.55	*21.65*	*1.08*	2.7	*2.6*	*0.1*			
**Th**	ppm	436	*451*	*23*	112.5	*117.5*	*6.0*	1.33	*1.29*	*0.09*			
**Tm**	ppm	3.33	*3.50*	*0.18*	2.77	*3.08*	*0.16*	2.29	*2.30*	*0.12*			
**U**	ppm	24.2	*24.1*	*1.2*	106.0	*111.8*	*5.6*	0.93	*0.80*	*0.07*			
**V**	ppm	375	*373*	*21*	1120	*1118*	*59*	8	*11*	*4*			
**Y**	ppm	402	*411*	*21*	294	*321*	*16*	123.0	*119.0*	*6.3*			
**Yb**	ppm	17.20	*17.00*	*0.88*	14.30	*15.50*	*0.80*	16.10	*14.80*	*0.75*			
**Zr**	ppm	1170	*1118*	*56*	953	*970*	*50*	609	*583*	*30*			

Supplementary Table 4. Rheological and thermal parameters of numerical models.

**Parameter**	**Units**	**Upper Crust**	**Lower Crust**	**Lithospheric Mantle**	**Asthenospheric Mantle**
Density	kg m^{-3}	2700	2850	3280	3300
Thermal expansivity	10^{-5} K^{-1}	2.7	2.7	3.0	3.0
Heat capacity	J $kg^{-1}K^{-1}$	1200	1200	1200	1200
Thermal diffusivity	10^{-7} m^2 s^{-1}	7.72	7.31	8.38	8.33
Radiogenic heat production	μW m^{-3}	0.8	0.2	0.0	0.0
Initial friction coefficient	-	0.5	0.5	0.5	0.5
Cohesion	MPa	5.0	5.0	5.0	5.0
Rheology		Wet Quartzite	Wet Anorthite	Dry Olivine	Dry Olivine
Pre-exponential constant	$Pa^{-n}s^{-1}$	$8.57e^{-28}$	$7.13e^{-18}$	$6.52e^{-16}$	$6.52e^{-16}$
Power law exponent	-	4.0	3.0	3.5	3.5
Activation energy	kJ mol^{-1}	223	345	530	530
Activation volume	cm^3 mol^{-1}	0	38	18	18
Flow law reference		Rutter & Brodie[39]	Rybacki et al.[40]	Hirth & Kohlstedt[41]	Hirth & Kohlstedt[41]

Supplementary References

1. Saria E., Calais E., Stamps D.S., Delvaux D., Hartnady C.J.H. (2014). Present-day kinematics of the East African Rift. *J. Geophys. Res.: Solid Earth*, 119, 3584–3600.

2. Ebinger C.J., Yemane T., Harding D.J., Tesfaye S., Kelley S., Rex D.C. (2000). Rift deflection, migration, and propagation: Linkage of the Ethiopian and Eastern rifts, Africa. *Geol. Soc. Am. Bull.*, 112(2), 163–176.

3. Brune S., Corti G., Ranalli G. (2017). Controls of inherited lithospheric heterogeneity on rift linkage: Numerical and analogue models of interaction between the Kenyan and Ethiopian rifts across the Turkana depression. *Tectonics*, 36, 1767-1786.

4. Benoit M.H., Nyblade A.A., Pasyanos M.E. (2006). Crustal thinning between the Ethiopian and East African plateaus from modeling Rayleigh wave dispersion. *Geophys. Res. Lett.*, 33(13), L13301.

5. Sippel J., Meeßen C., Cacace M., Mechie J., Fishwick S., Heine C., Scheck-Wenderoth M., Strecker M.R. (2017). The Kenya rift revisited: insights into lithospheric strength through data-driven 3-D gravity and thermal modelling. *Solid Earth*, *8*(1), 45–81.

6. Bosworth W. (1992). Mesozoic and Early Tertiary rift tectonics in East Africa. *Tectonophysics*, 209, 115-137.

7. Vétel W., Le Gall B., Walsh J.J. (2005). Geometry and growth of an inner rift fault pattern: the Kino Sogo Fault Belt, Turkana Rift (North Kenya). *J. Struct. Geol.*, 27, 2204–2222.

8. Vétel W., Le Gall B. (2006). Dynamics of prolonged continental extension in magmatic rifts: the Turkana Rift case study (North Kenya). *Geol. Soc. Lond. Spec. Publ.*, 259, 209–233.

9. Boone S.C., Seiler C., Kohn B.P., Gleadow A.J.W., Foster D.A., Chung L. (2018). Influence of rift superposition on lithospheric response to East African Rift System extension: Lapur Range, Turkana, Kenya. *Tectonics*, 37, 182-207.

10. Brotzu P., Morbidelli L., Nicoletti M., Piccirillo E. M., Traversa G. (1984). Miocene to Quaternary volcanism in eastern Kenya: sequence and geochronology. *Tectonophysics*, 101(1-2), 75-86.

11. Morley C.K., Wescott W.A., Stone D.M., Harper R.M., Wigger, S.T., Day R.A. Karanja, F.M. (1999). Geology and geophysics of the Western Turkana basins, Kenya. In C. K. Morley (Ed.), Geoscience of rift systems—Evolution of East Africa. *AAPG studies in geology*, 44, 19–54.

12. McDougall I., Brown F.H. (2009). Timing of volcanism and evolution of the northern Kenya Rift. *Geol. Mag.*, 146, 34–37.

13. Shinjo R., Chekol T., Meshesha D., Itaya T., Tatsumi Y. (2011). Geochemistry and geochronology of the mafic lavas from the southeastern Ethiopian rift (the East African Rift System): assessment of models on magma sources, plume–lithosphere interaction and plume evolution. *Contrib. Mineral. Petrol.*, 162(1), 209-230.

14. Macgregor D. (2015). History of the development of the East African Rift System: A series of interpreted maps through time. *J. Afr. Earth Sci.*, 101, 232–252.

15. Boone S.C., Kohn B.P., Gleadow A.J.W., Morley C.K., Seiler C., Foster D.A., Chung L. (2018). Tectono-thermal evolution of a long-lived segment of the East African Rift System: Thermochronological insights from the North Lokichar Basin, Turkana, Kenya. *Tectonophysics*, 744, 23–46.

16. Kim Y.-S., Sanderson D.J. (2005). The relationship between displacement and length of faults: a review. *Earth Sci. Rev.*, 68, 317-334

17. Walsh J.J., Nicol A., Childs C. (2002). An alternative model for the growth of faults. *J. Struct. Geol.*, 24, 1669–1675.

18. Corti G. (2009). Continental rift evolution: from rift initiation to incipient break-up in the Main Ethiopian Rift, East Africa. *Earth Sci. Rev.*, 96, 1–53.

19. Philippon M., Corti G., Sani F., Bonini M., Balestrieri M.L., Molin P., Willingshofer E., Sokoutis D., Cloetingh S. (2014). Evolution, distribution and characteristics of rifting in southern Ethiopia. *Tectonics*, 33, 485-508, doi:10.1002/2013TC003430.

20. Wilson B.M. (2007). Igneous petrogenesis a global tectonic approach. Springer Science & Business Media, 466 pp.

21. Le Bas M., Maitre R.L., Streckeisen A., Zanettin B., IUGS Subcommission on the Systematics of Igneous Rocks. (1986). A chemical classification of volcanic rocks based on the total alkali-silica diagram. *J. Petrol.*, 27(3), 745-750.

22. Leeder M.R., Gawthorpe R.L. (1987). Sedimentary models for extensional tilt-block/half-graben basins. In M.P. Coward, J.F. Dewey and P.L. Hancock (Eds.), Continental extensional tectonics. *Geol. Soc. Lond. Spec. Publ.*, 28, 139-152.

23. Charsley, TJ. (1987). Geology of the North Horr area. *Kenya Mines Geol. Dep., Rep.*, 110: 40 pp.

24. Charsley, TJ. (1988). Geological Map of Kenya - Degree sheet 12, North Horr (scale 1:250,000). Government of Kenya, Ministry of Environment and Natural Resources, Mines and Geological Department, Nairobi (Kenya).

25. Morley C.K., Wescott W.A., Stone D.M., Harper R.M., Wigger S.T., Karanja F.M. (1992). Tectonic evolution of the northern Kenyan Rift. *J Geol Soc, London*, 149, 333-348.

26. Bull W.B., McFadden, L. (1977). Tectonic Geomorphology of North and South of the Garlock Fault, California. In: Dohring, D.O., Ed., Geomorphology in Arid Regions, Publ. in Geomorphology, State University of New York, Binghamton, 115-138.

27. Viste E., Korecha D., Sorteberg A. (2013). Recent drought and precipitation tendencies in Ethiopia. *Theor. Appl. Climatol.*, 112 (3-4), 535-551

28. William A.P., Funk C., Michaelsen J., Rauscher S.A., Robertson I., Wils T.H.G., Koprowski M., Eshetu Z., Loader N.J. (2012). Recent summer precipitation trends in the Greater Horn of Africa and the emerging role of Indian Ocean sea surface temperature. *Clim. Dyn.*, 39 (9-10), 2307-2328.

29. Keller E.A. (1986). Investigations of active tectonics: Use of surficial earth processes. In: Active Tectonics, National Academy Press, Washington, D. C., 136-147.

30. Harvey A.M. (2002). Effective timescales of coupling within fluvial systems. *Geomorphology*, 44, 175–201.

31. Viseras C., Calvache M.L., Soria J.M., Fernández J. (2003). Differential features of alluvial fans controlled by tectonic or eustatic accommodation space. Examples from the Betic Cordillera, Spain. *Geomorphology*, 50, 181–202.

32. Irvine T.N., Baragar W.R.A. (1971). A Guide to the Chemical Classification of the Common Volcanic Rocks. *Can. J. Earth Sci.*, 1971, 8(5): 523-548, https://doi.org/10.1139/e71-055.

33. Orlando A., Abebe T., Manetti P., Santo AP, Corti G. (2006). Petrology of mantle xenoliths from Megado and Dilo, Kenya rift, southern Ethiopia. *Ofioliti*, 31(2), 71-87.

34. Furman T., Bryce J.G., Karson J., Iotti A. (2004). East African Rift System (EARS) plume structure: insights from Quaternary mafic lavas of Turkana, Kenya. *J. Petrol.*, 45(5), 1069-1088.

35. Class C., Altherr R., Volker F., Eberz G., McCulloch M.T. (1994). Geochemistry of Pliocene to Quaternary alkali basalts from the Hurri Hills, northern Kenya. *Chem. Geol.*, 113(1-2), 1-22.

36. Bruhn R. L., Brown F.H., Gathogo P.N., Haileab B. (2011). Pliocene volcano-tectonics and paleogeography of the Turkana Basin, Kenya and Ethiopia. *J. Afr. Earth Sci.*, 59(2-3), 295-312.

37. Mazzarini F., Le Corvec N., Isola I., Favalli M. (2016). Volcanic field elongation, vent distribution, and tectonic evolution of a continental rift: The Main Ethiopian Rift example. *Geosphere*, 12, 706–720, doi:10.1130/GES01193.1.

38. Le Maitre, R. W., Streckeisen, A., Zanettin, B., Le Bas, M. J., Bonin, B., & Bateman, P. (Eds.). (2005). Igneous rocks: a classification and glossary of terms: recommendations of the International Union of Geological Sciences Subcommission on the Systematics of Igneous Rocks. Cambridge University Press.

39. Rutter E.H., Brodie K.H. (2004). Experimental grain size-sensitive flow of hot-pressed Brazilian quartz aggregates. *J. Struct. Geol.*, 26, 2011–2023, doi: 10.1016/j.jsg.2004.04.006.

40. Rybacki E., Gottschalk M., Wirth R., Dresen G. (2006). Influence of water fugacity and activation volume on the flow properties of fine-grained anorthite aggregates. Journal of Geophysical Research. *Solid Earth*, 111, B03203, doi: 10.1029/2005JB003663.

41. Hirth G., Kohlstedt D.L. (2003). Rheology of the upper mantle and the mantle wedge: A view from the experimentalists. *Geophysical Monograph*, 138, 83–105.
